# A novel biostimulant obtained from red wine lees enhances tolerance to ozone-induced abiotic stress in grapevine plants

**DOI:** 10.3389/fpls.2026.1654494

**Published:** 2026-02-03

**Authors:** Emilia Naranjo, Angel Orts, José M. Orts, Luis Martin-Presas, Angélica Castaño, Juan Parrado

**Affiliations:** Department of Biochemistry and Molecular Biology, Facultad de Farmacia, Universidad de Sevilla, Sevilla, Spain

**Keywords:** grapevine, ozone, red wine lees, ROS, transcriptomic

## Abstract

The main objective of this study was to investigate the ability of a novel plant biostimulant obtained from red wine lees by enzymatic hydrolysis (LEE) to protect against abiotic ozone damage in grapevine plants (*Vitis vinifera*). Tropospheric ozone at high concentrations is considered a pollutant that harms plants by entering through the leaves via the stomata. Once inside, it decomposes into reactive oxygen species (ROS), causing oxidative stress, reduced photosynthesis, growth alterations, and visible symptoms such as necrotic spots and yellowing. LEE counteracted ozone-induced damage, as evidenced by net photosynthetic rate, electron transport rate, effective quantum yield of Photosystem II, and delayed fluorescence. LEE triggered the overexpression of genes involved in the synthesis of secondary metabolites that reduce oxidative damage and enhance resilience. Additionally, it decreases energy expenditure on emergency responses, as shown by the lower expression of stress-related genes. Consequently, the biostimulant optimizes metabolic efficiency and plant health.

## 1 Introduction

Ozone, an allotrope of oxygen, forms in the troposphere through complex chemical reactions between nitrogen oxides and volatile organic compounds, triggered by sunlight. This process is particularly pronounced in urban and industrial areas, where increasing human activity leads to a steady rise in tropospheric ozone levels (Wilkinson et al., 2012). Recognized as a major air pollutant, tropospheric ozone has detrimental effects on plant growth and productivity (Betzelberger et al., 2012; Wilkinson et al., 2012). The extent of ozone impact on plants depends on its concentration and the duration of exposure. Long-term exposure to low ozone levels reduces photosynthesis, stunts growth, and accelerates aging, often without visible damage to plant tissues. In contrast, short-term exposure to high ozone concentrations—an event that can occur multiple times during the growing season in various regions—triggers cell death and the formation of visible lesions in ozone-sensitive plants (Agathokleous et al., 2023; Nowroz et al., 2023). The grapevine (*Vitis vinifera, L*.) are sensitive to ozone, and elevated ozone levels can impair their photosynthetic performance, growth, yield, and fruit quality (Fumagalli et al., 2019).

The introduction of new legislation on recycling and the sustainable use of underutilized local resources in many countries has highlighted the importance of the circular bioeconomy for the manufacturing of high-value products (Spekreijse et al., 2019). Wine is one of the most widely produced alcoholic beverages globally, with an approximate production of 259.9 million hL in 2022. Typical wastes and by-products from wineries include grape pomace (composed of skins and seeds, which represent about 60% of total winemaking by-products, and stems, which account for approximately 14%), as well as grape solids and fermentation (yeast) lees (26%). Each year, an estimated seven million tons of grape pomace and lees are generated worldwide (Bordiga et al., 2019).

In recent years, there is a growing interest in reusing these food wastes not only to reduce their environmental impact by the circular production of by-products but also to entail an economic benefit derived from the reuse of products with added value (Baroi et al., 2022; Dwyer et al., 2014). Given a circular economy approach, some of these wastes can be successfully “recycled”, reused, or recovered, improving both the economic and environmental. These by-products are typically used for animal feeding, composting, industrial biomass, or distillate production (Bordiga et al., 2015). However, grape pomace and lees are high added-value by-products due to its wide variety of compounds. The wine lees are a combination of yeasts, organic acids, insoluble carbohydrates, inorganic salts, lignin, proteins, phenolic compounds, and ethanol (Jara-Palacios, 2019). These compounds are susceptible of extraction or transformation and exploitation with the consequent economic benefit.

Biostimulants are currently regarded as key tools in sustainable agriculture because they enhance specific plant functions that ultimately support crop performance under suboptimal conditions. In functional terms, plant biostimulants are products applied in small amounts that modulate plant physiological and biochemical processes, thereby improving nutrient use efficiency, activating or priming defense pathways, and increasing tolerance to abiotic stresses while contributing to yield and quality improvements (Russo and Berlyn, 1991; Yakhin et al., 2017). Historically, before the term “biostimulant” became widespread, expressions such as “biogenic stimulators” or “biogenic stimulants” were used for compounds produced in plant tissues under non-lethal stress that stimulated vital physiological reactions (Yakhin et al., 2017). The early concept of “organic plant biostimulants” proposed by Russo and Berlyn already emphasized their non-nutritional nature and their ability to enhance productivity and stress resistance in low-input systems (Russo and Berlyn, 1991). In line with recent literature and the current EU Fertilising Products Regulation (European Union, 2019), as well as related regulatory frameworks in other regions, biostimulants are now primarily defined from their agronomical function rather than their composition, focusing on their capacity to improve nutrient use efficiency and to prime plant defense and stress-response pathways, which in turn supports higher resilience and better crop performance.

In our group, we have previously developed microbiological and enzymatic technologies that allows obtaining biostimulants from agro-industrial residues, both of plant origin such as okara (García-Martínez et al., 2010; Orts et al., 2019, 2024) or rice bran (Macias-Benitez et al., 2021), and of animal origin such as chicken feathers (Caballero et al., 2020; Rodríguez-Morgado et al., 2014) or even from sewage sludge (Caballero et al., 2022; Rodríguez-Morgado et al., 2015; Tejada et al., 2013).

Considering the relevance of the wine industry in Mediterranean countries such as Spain, Greece, and Italy, regions with high tropospheric ozone levels, there is an emerging interest in developing protection strategies against ozono-induced oxidative damage (Doche et al., 2014; Wang et al., 2024). Such strategies are particularly valuable when they avoid environmental toxicity, as is the case with plant-derived extracts. In this context, the enzymatic technology developed by our group offers a dual advantage: it converts viticulture wastes into biostimulants aligned with circular economy principles, while simultaneously providing a sustainable tool to enhance plant resilience against ozone stress. This study specifically evaluates the protective role of these biostimulants in mitigating ozone-induced oxidative damage, bridging waste valorization with agroecological protection strategies.

## 2 Materials and methods

### 2.1 Lees enzymatic extract preparation

Agro-industrial waste from the wine industry wastes was obtained from the winery “Cooperativa Nuestra Señora del Socorro” (Rociana del Condado, Huelva). The lees (diluted 15% in water) were processed by enzymatic hydrolysis using subtilisin (0.3% v/v) (EC 3.4.21.62), a protease from *Bacillus licheniformis* (Biocom Española S.A; protease activity 650±5% UB/g, 1.110-1.120 g/ml, as stated in the technical datasheet), as hydrolytic agents in a bioreactor with controlled temperature (55°C) and pH (pH 9), using the pH-stat method (Parrado et al., 2006).

In addition, enzymatic biostimulant production was monitored by studying the performance of the process as a function of the evolution of the extraction of soluble hydrolyzed biomolecules as peptides, carbohydrates and polyphenols.

### 2.2 Chemical characterization of lees enzymatic extract

The chemical characterization of the products obtained has been carried out. Previously, these products were freeze-dried using the Telstar Cryodos -80°C equipment (Spain).

#### 2.2.1 Analysis of free amino acids using the high-resolution chromatographic system UPLC ACQUITY I-CLASS

The lyophilized samples were extracted following the protocol described in Kıvrak (2015). Briefly, 100 mg were weighed into a 2 mL Eppendorf tube, and 1 mL of MeOH/H_2_O (80:20) (v/v) with 0.1% HCOOH was added. The mixture was sonicated for 5 minutes, vortexed afterwards, and then immediately centrifuged at 4°C at 4,000 rpm for 15 minutes. The supernatant was filtered through a 0.22 µm pore-size PVDF membrane filter. Subsequently, it was injected into the UPLC system equipped with a binary pump and an autosampler for up to 96 vials with cooling. The chromatographic conditions tested were based on previous experiences of the Metabolomics Service of CEBAS-CSIC (Murcia) for this type of analysis.

#### 2.2.2 Analysis of the organic extracts using the high-resolution chromatographic system UPLC coupled to mass spectrometry

The organic extracts were extracted following the protocol described by Maren Müller and Sergi Munné-Bosch (Müller and Munné-Bosch, 2011), and were then injected into the UPLC system equipped with a binary pump and an autosampler for up to 96 wells with cooling. The chromatographic conditions used were based on this protocol This assay was carried out at the Metabolomics Service of CEBAS-CSIC (Murcia).

#### 2.2.3 Extraction of primary (polar/semipolar) metabolites using ¹H-NMR spectroscopy

This assay was carried out at the Metabolomics Service of CEBAS-CSIC (Murcia) following the method previously described (Choi et al., 2004, 2006).

#### 2.2.4 Antioxidant capacity

The evaluation of the antioxidant capacity of the lees enzymatic extract was evaluated using the PPPH (Brand-Williams et al., 1995; Xiao et al., 2020), ABTS assays (Kotha et al., 2022; Re et al., 1999). The phenolics content was determined using the Folin-Ciocalteu methods by reacting the extracts with the homonymous reagent (Simões et al., 2024; Singleton et al., 1974).

### 2.3 Plants treatment

The selected plants and the treatment applied were carried out according to previous work by the group (Macias-Benitez et al., 2021). *Vitis vinifera* cv. Syrah plants were cultivated from seedlings purchased from the Barber nursery (L’Olleria, Valencia, Spain) in plastic pots containing an organic commercial substrate (Gramofor GmbH und Co. KG.) and Osmocote^®^ (NPK 15: 9: 12), and grown inside the University of Seville Glasshouse General Services on a phytoclimatic chamber, Fitoclima 18,000 PHL (AralabSpain), with a controlled temperature of 18−22°C, 50% relative humidity, adequate irrigation with tap water and a photoperiod of 16 h light/8 h darkness, with a maximum photosynthetic photon flux density incident on the leaves of 1200 μmol m^−2^ s^−1^.

After eight days of transplantation, 20 plants were selected and divided in 4 groups (5 plants for group): control plants (group Ct), control plants under ozone (group O_3_) exposition (group Ct+ O_3_), plants treated with LEE (group LEE) and plants treated with LEE under O_3_ exposition (LEE+ O_3_). Following protocol previously describe by us (Macias-Benitez et al., 2021), to evaluate the protection capacity of the treatment with LEE, plants were foliar sprayed a total of 4 times at five-day intervals, with an aqueous solution of LEE at 0.1% (groups LEE and LEE+ O_3_) or distilled water (groups Ct and Ct+ O_3_). After 5 days of the last spray treatment, Ct+ O_3_ and LEE+ O_3_ plants were transferred to another phytoclimatic chamber with an ozone generator (ZONOSISTEM GM 5000 O_3_ Generator) attached and exposed to 3 consecutive fumigations with 300 ppb of O_3_ for 6 h (from 10:00 am to 4:00 pm). After ozone fumigation all the test plants were sprayed again with the corresponding solution (LEE 0.1% or distilled water). An acute ozone exposure of 300ppb was selected to induce a clear and reproducible oxidative stress and visible leaf damage in a short time, to evaluate the protective effect of the enzymatic extract. This concentration is higher than typical environmental ozone levels and is used here as a controlled stress test rather than a simulation of chronic air pollution.

Finally, 24 h after the last exposure to ozone, foliar samples were taken from each plant and the analyses described below were carried out.

### 2.4 Plants status after the ozone exposition

#### 2.4.1 Analyses of photosynthetic parameters

Twenty-four hours after the last ozone treatment, net photosynthetic rate (A_N_), electron transport rate (ETR), effective quantum yield of PSII (PhiPS2) and intercellular CO_2_ concentration (Ci) were determined in plants using an IRGA (LI-6400XT, LI-COR Inc., Nev., EEUU) with a light chamber for the leaf (Li-6400-02B, Li-Cor Inc.) according to Macias-Benitez et al. (2021). Briefly, measurements (n = 20) were performed between 10 a.m. and 2 p.m. hours under a photosynthetic photon flux density of 1500 \upmu mol.m^−2^. s^−1^, a deficit of vapor pressure of 2–3 kPa, a temperature of approximately 25°C, and an ambient CO_2_ concentration of 400 \upmu mol.mol^−1^ air. Each measurement was recorded after gas exchange had stabilized (120 s).

#### 2.4.2 Delayed fluorescence determination

Delayed fluorescence (DF) was recorded at the end of the experiment in a random leaf from each plant. For that, the collected leaves were analyzed using a NightShade LB 985 (Berthold Technologies, Germany) equipped with a deeply cooled CCD camera (López-Jurado et al., 2020). The recorded data were converted to counts per second (cps) and normalized to the leaf area.

#### 2.4.3 Statistical analysis

To assess the physiological state of the plants, the statistical analysis was carried out with SigmaPlot software (version 12.0, Systat Software Inc., San José, CA, USA). Normality and homogeneity of variances were tested, and results were compared using ANOVA followed by Tukey’s test (p<0.05) to determine significant differences among treatments.

### 2.5 Analyze gene expression in plants: RNAseq

#### 2.5.1 Sample collection

The sample collection was carried out following the protocol provided by the company Corning.

#### 2.5.2 Extraction, purification of samples and library preparation

The extraction and purification of the input RNA was performed by GENEWIZ Multiomics & Synthesis Solutions from Azenta Life Sciences.

#### 2.5.3 Mapping sequence reads to the reference genome

Sequence reads were trimmed to remove possible adapter sequences and nucleotides with poor quality using Trimmomatic v.0.36. The trimmed reads were mapped to the Vitis_vinifera_GCA_030704535.1 reference genome available on ENSEMBL using the STAR aligner v.2.5.2b. The STAR aligner is a splice aligner that detects splice junctions and incorporates them to help align the entire read sequences. BAM files were generated as a result of this step. The raw sequencing data (fastq files) have been deposited in the NCBI Sequence Read Archive and can be accessed under BioProject accession *PRJNA1377885*.

#### 2.5.4 Extracting gene hit counts

Unique gene hit counts were calculated by using feature Counts from the Subread package v.1.5.2. The hit counts were summarized and reported using the gene_id feature in the annotation file. Only unique reads that fell within exon regions were counted.

#### 2.5.5 Differential gene expression analysis

After extraction of gene hit counts, the gene hit counts table was used for downstream differential expression analysis. Using DESeq2, a comparison of gene expression between the customer-defined groups of samples was performed. The Wald test was used to generate p-values and log2 fold changes. Genes with an adjusted p-value < 0.05 and absolute log2 fold change > 1 were called as differentially expressed genes for each comparison.

#### 2.5.6 Bioinformatics tools for functional analysis

To verify the annotation, and thus the function of the overexpressed genes and proteins, the gene ontology provided by UniprotKB, annotations from NCBI, PATRIC, and Ecogene were consulted, as well as the gene ontology assigned by the JCVI Microbial Resource Center. Additionally, these genes and proteins were sorted according to the orthologous classification provided by KEGG (Kanehisa et al., 2008), incorporating into this classification those genes and proteins reviewed by the various annotations and ontologies mentioned earlier. As the first functional analysis, the different functional categories described in the clusters of orthologous groups (COG) associated with each overexpressed gene or protein were consulted.

## 3 Results

### 3.1 Extraction and characterization process of the LEE

The composition of the lees makes it an interesting product to be used as a biostimulant. To fully exploit its properties, it is necessary to modified the insoluble fraction and make it more available. To achieve this goal, enzymatic hydrolysis has been proposed using the enzyme subtilisin. This protease extracts, solubilizes, and hydrolyzes the initial proteins into soluble peptides, and also leads to the solubilization of hydrophobic compounds such as lipids and bioactive metabolites. The enzymatic hydrolysate showed an approximate increase of 13% in the soluble fraction compared to the control without enzymes, reaching up to a 38% total solubilization. The biostimulant´s characterization is shown in **Table 1**.

**Table 1:** Analytical composition of LEE (red wine lees by enzymatic hydrolysis).

	% PROT (Nx5,75)		FT (mg GAE/g dry matter)		DPPH (µmoles TE/g dry matter)		ABTS (µmoles TE/g dry matter)	
Samples	MEDIA	SD	MEDIA	SD	MEDIA	SD	MEDIA	SD
Red lees	32,34	0,98	37,88	3,49	244,53	10,01	362,84	43,60
LEE	39,73	1,54	202,87	48,51	624,11	59,52	2235,14	235,71

FT, total phenols; DPPH, 1, 1-diphenyl-2-picrylhydrazyl radical; ABTS, 2,2’-azino-bis-3-ethylbenzothiazoline-6-sulfonic acids. SD, standard deviation.

As shown in the **Table 1**, the hydrolyzed product (LEE) showed approximately 19% more protein than the raw material, and about 6 times more total phenols compared to the red lees, which is very important data since phenols are fundamental in plant growth processes and help responses to environmental stress.

Regarding antioxidant capacity, this was measured using two different methods, DPPH and ABTS, with the observation that the antioxidant power is greater in the hydrolyzed product than in the raw material, being 6 times higher in ABTS and 2.5 times higher in DPPH (**Table 1**).

Next, the metabolites contained in the biostimulant were analyzed using high-resolution UPLC chromatography and ¹H-NMR spectroscopy. As shown in **Table 2**, the hydrolysate of red lees (LEE) provided the essential amino acids that plants cannot synthesize and therefore must obtain from external sources (Sun et al., 2024), such as threonine, valine, isoleucine, and leucine. Proline stood out as the most abundant amino acid, an important fact because, along with glycine-betaine, it is considered an osmoprotective amino acid against various stresses (Hosseinifard et al., 2022).

**Table 2:** Metabolites composition of LEE.

Metabolites µg/100 mg	Samples	LEE
Aminoacids	GABA	163,31
	Alanine	246,23
	Arginine	344,97
	Glutamate	201,09
	Glutamine	261,23
	Isoleucine	211,68
	Leucine	636,96
	Proline	1489,16
	Threonine	ND
	Valine	219,29
Organic Acids	Acetate	404,19
	Citrate	276,54
	Formate	21,57
	Fumarate	4,29
	Lactate	1353,53
	Malate	216,39
	Succinate	501,97
	Tartrate	17361,99
Sugars	Fructose	1610,75
	Glucose	943,84
	Sucrose	440,94
Other	Glycerol	4843,46

N.D., not determined.

The most representative organic acids were tartrate and lactate. These results are completely expected, as the hydrolysate of red wine lees comes from a complete winemaking process (including malolactic fermentation), which can provide beneficial properties to the biostimulant. Regarding sugars, both fructose and glucose are the reducing sugars naturally present in grapes and must; therefore, it is normal for their levels to be much higher than that of sucrose, which is not the primary sugar in grapes.

### 3.2 Physiological status in plants

**Figure 1:**
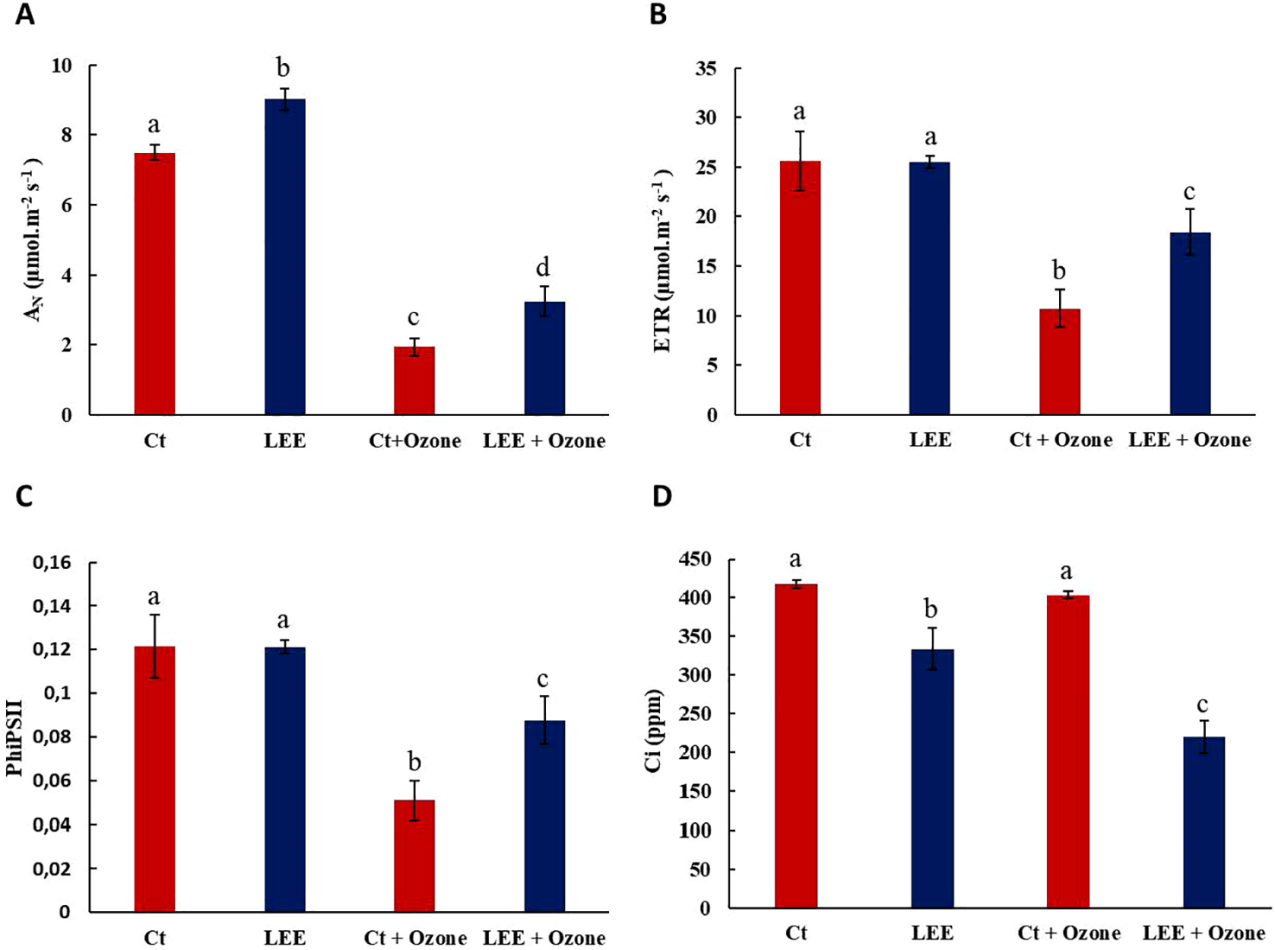
Physiological parameters. **(A)** A_N_; **(B)** ETR; **(C)** PhiPSII and **(D)** Ci in response to O_3_ (0 and 300 ppb) under a treatment without and with LEE. Values represent mean ± standard deviation (SD, n = 5). Different letters indicate means that are significantly different from each other (one-way ANOVA, Ozone treatment; HSD test, P<0.05). A_N_, Net CO_2_ assimilation rate; ETR, Electron Transport Rate; PhiPSII, Effective quantum yield of Photosystem II and Ci, Intercellular CO_2_ concentration; Ct, control plants; LEE, Plants treated with biostimulant; Ct+Ozone, plants with ozone and LEE +Ozone, plants treated with ozone and biostimulant.

The physiological state of the plants was determined through the parameters A_N_, ETR, PhiPSII and Ci. Ozone exposure (300 ppb) significantly affected these parameters (**Figures 1A-D**), reducing them by 74%, 58%, and 57%, respectively, compared to the control, except for the parameter Ci, which showed no effect. Although the 300 ppb ozone treatment used in this study corresponds to relatively high ambient ozone concentrations, it was chosen as an acute stress test to clearly demonstrate the protective capacity of the enzymatic extract.

The treatment with the biostimulant LEE did not change the physiological parameters ETR and PhiPSII compared to the control plants, however, it increased the photosynthetic rate by 20% and reduced the Ci parameter by 19%. Protection against the O_3_-induced decrease was observed in the photosynthetic rate, electron transport rate and effective quantum yield of photosystem II (40% in A_N_; 42% in ETR; 41% in PhiPSII) (**Figures 1A–C**). Conversely, the intercellular CO_2_ concentration decreased by 45% with the application of LEE compared to the ozone-treated control (**Figure 1D**).

**Figure 2:**
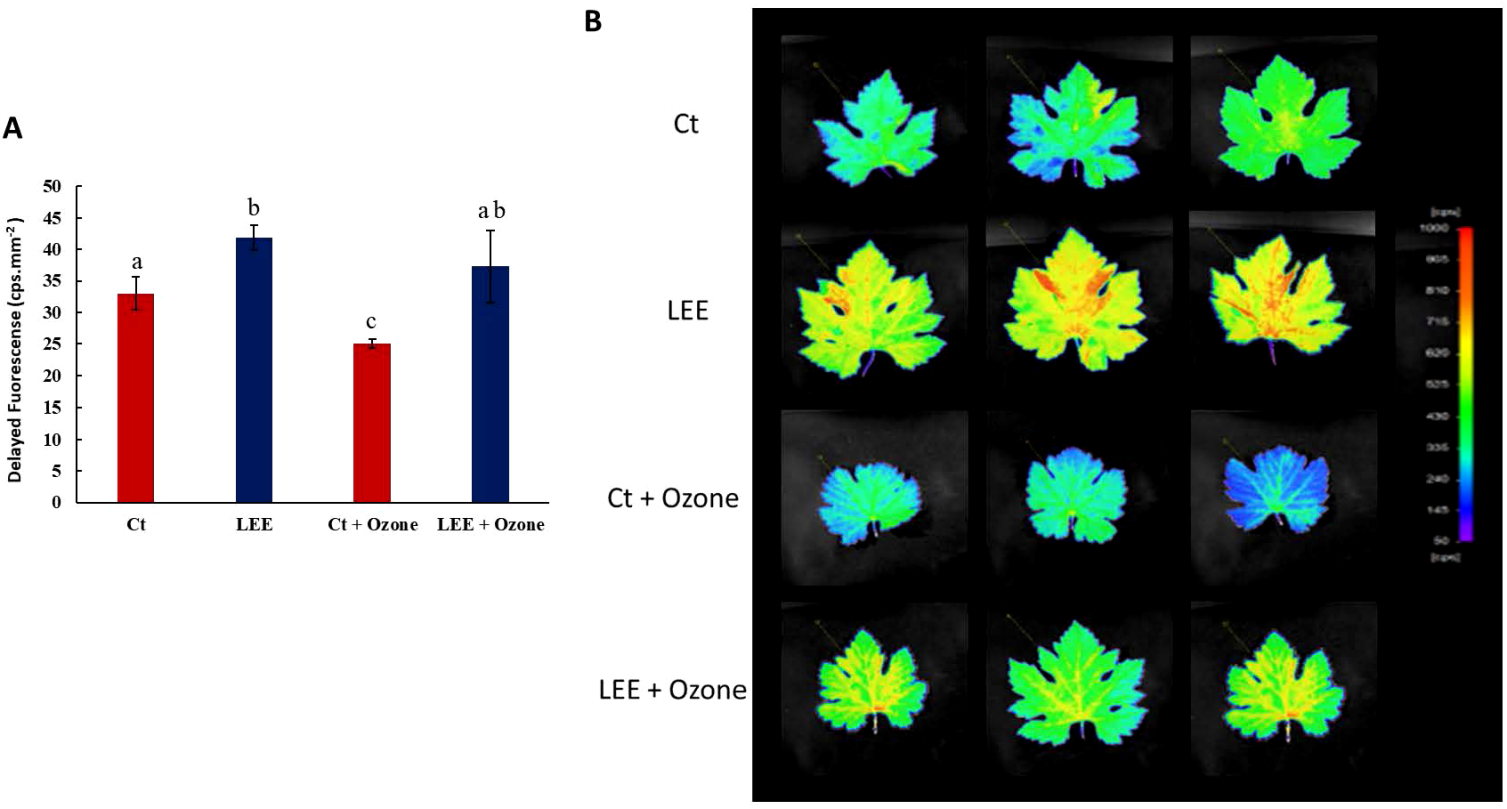
Delayed fluorescence in leaves of Vitis vinifera plants in response to ozone (O3) (0 and 300 ppb) under a treatment without and with LEE). **(A)** Counts per second (cps) values for each treatment. Values represent mean ± standard deviation (SD, n = 5). Different letters indicate means that are significantly different from each other (one-way ANOVA, Ozone treatment; HSD test, P<0.05). Ct: control plants; LEE: Plants treated with biostimulant; Ct+Ozone: plants with ozone and LEE +Ozone: plants treated with ozone and biostimulant. **(B)** photographs taken by the plant imaging system NightShade LB 985. Delayed fluorescence was used as a direct indicator of the chlorophyll content. The color scale reflects the detected counts per second (cps) of delayed fluorescence emission in leaves. Red color indicates high intensities representing high chlorophyll content, blue color indicated low intensities of fluorescence, indicating low amounts of chlorophyll. Ct, control plants; LEE, Plants treated with biostimulant; Ct+Ozone, plants with ozone and LEE +Ozone, plants treated with ozone and biostimulant.

Next, the delayed fluorescence parameter was studied. This phenomenon is an indicator of the plant’s health since the intensity of delayed fluorescence is an indicator of chlorophyll content and the physiological state of the plant. It also allows for the evaluation of plant responses to stress factors such as drought, salinity, infections, thermal shock, and heavy metal contamination (Goltsev et al., 2009). As could be observed in **Figure 2**, (graph **Figures 2A**, **2B**), application of LEE increased the delayed fluorescence values by approximately 27% compared to the control. Exposure to ozone significantly decreased fluorescence values (25% compared to the control), and this decrease was completely prevented by LEE.

These results suggest that LEE improves photosynthetic activity and the overall physiological state of the plant, and also protects against ozone-induced photosynthetic damage as it maintains the physiological state induced by this stress.

### 3.3 Transcriptional analysis of *Vitis vinifera* genes in the presence of ozone and LEE

#### 3.3.1 Effect of ozone (300 ppb) on *Vitis vinifera* plants

Exposure to high concentrations of ozone causes visible lesions, including spots, chlorosis, and necrosis, among others (Feng et al., 2015; Tiwari and Agrawal, 2018). However, it also induces other less apparent injuries, such as the degradation of palisade mesophyll cells, the accumulation of starch and lipids in leaf tissues, the deposition of callose and phenolic compounds in cell walls, and vacuoles filled with polyphenols and tannins (Agathokleous et al., 2015). From a physiological perspective, O_3_ exposure leads to a decrease in stomatal conductance, photosynthesis, and photochemical efficiency, along with an increase in induced dark respiration (Betzelberger et al., 2010; Biswas et al., 2008; Fiscus et al., 2005). These alterations result in gene deregulation, either upregulation or downregulation, remodeling of protein expression patterns, stimulation of defense pathways, and disruption of metabolic processes (Singh et al., 2023). The results of the statistical analysis are provided in the Supplementary Material (**Supplementary Figures S1**-**S5**).

**Figure 3:**
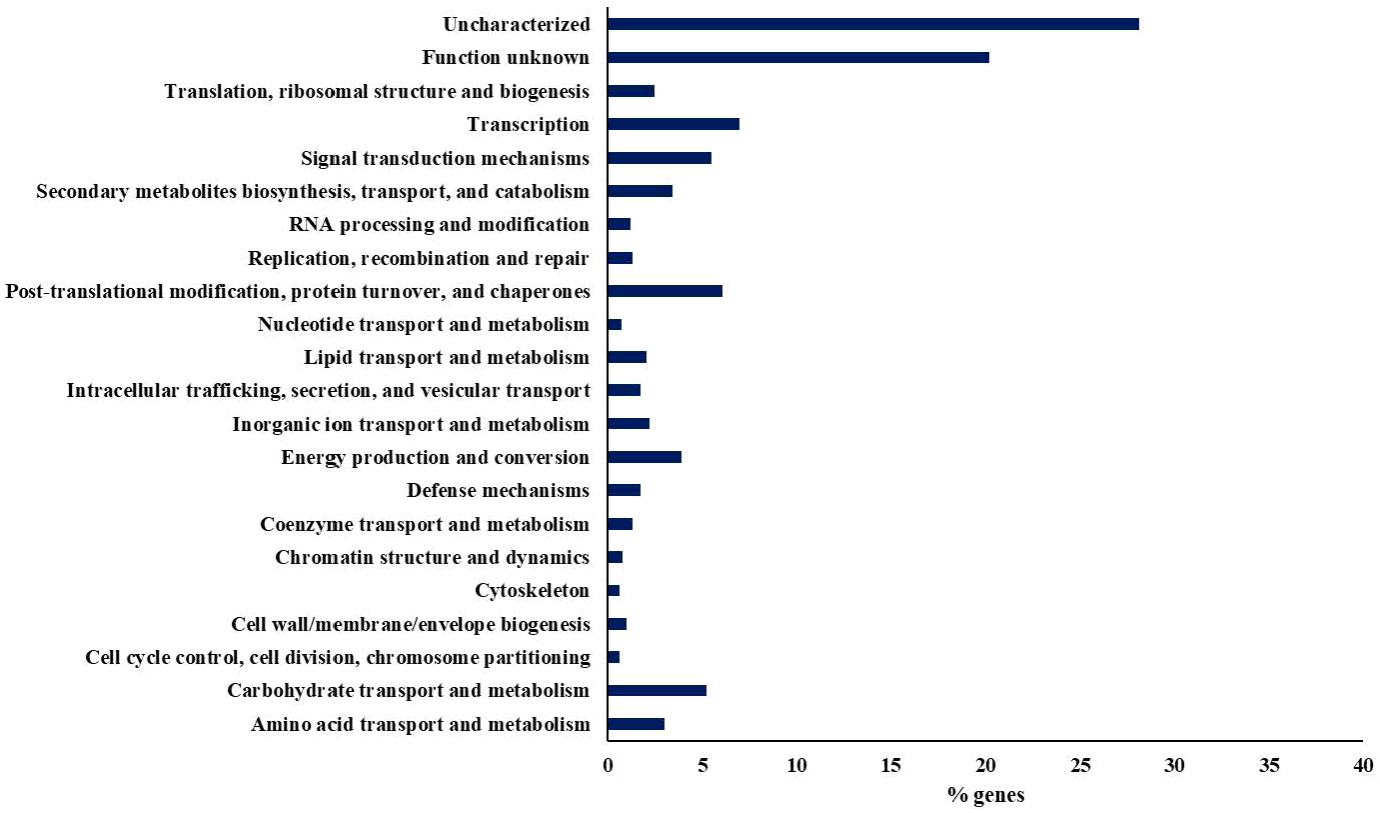
Distribution of the percentage of differentially expressed genes after exposure to 300 ppb of ozone classified into functional categories assigned by COG.

Ozone exposure caused 4,092 differentially expressed genes, of which 1,950 were downregulated and 2,142 were upregulated. First, an initial analysis of the differentially expressed genes was performed using the COG (“Cluster of Orthologous Groups”) classification (Tatusov et al., 2003) (**Figure 3**). The most representative functional categories were the transcription, post-translational modification, protein turnover, and chaperones, signal transduction mechanisms, carbohydrate metabolism and transport, and the catabolism, transport, and biosynthesis of secondary metabolites.

At the transcriptional level, transcription factors related to phytohormones, which are key in plant growth and development, are differentially expressed. These include AP2-like ethylene-responsive transcription factors, auxin response factors, and ethylene-responsive transcription factors. Additionally, various factors related to responses to abiotic stress were identified, such as BHLH domain-containing proteins (Basic Helix-Loop-Helix), and MYB, WRKY, and NAC transcription factors.

Regarding post-translational modification, protein turnover, and chaperones, a group of proteins known as chloroplastic protein DnaJ (VitviT2T_009334, VitviT2T_008751, VitviT2T_019324, VitviT2T_019456) stood out. These function as molecular chaperones, either alone or in association with heat shock proteins 70 (Hsp70), and participate in essential cellular processes such as protein folding, degradation, and refolding (Hennessy et al., 2005; Zhao et al., 2022). Additionally, they play a crucial role in maintaining photosystem II under cold stress (Kong et al., 2014). Our data have also revealed the overexpression of the enzyme Glutathione S-transferases, enzymes differentially expressed in response to abiotic stress signals (Csiszár et al., 2014; Hernández Estévez and Rodríguez Hernández, 2020).

In grapevine plants exposed to ozone, serine/threonine protein kinases were observed to be overexpressed as part of signal transduction systems. These proteins transmit extracellular signals to cellular responses by phosphorylating target proteins on serine or threonine residues.

Ozone has induced the overexpression of genes involved in the synthesis of biotin and phenolic compounds known as stilbenes, including resveratrol, in plants. These genes are the biotin synthase (VitviT2T_021744), an enzyme that catalyzes the addition of a sulfur atom to dethiobiotin, forming biotin. In addition, genes related to stilbene synthesis have also been found, with several key enzymes standing out, such as stilbene synthases (VitviT2T_025038, VitviT2T_025014, VitviT2T_006858) and trihydroxystilbene synthases (VitviT2T_009682, VitviT2T_011709, VitviT2T_025021, VitviT2T_025030, and VitviT2T_025033).

The high ozone concentration also led to the inhibition of 50S and 30S ribosomal proteins present in chloroplasts and mitochondria (**Table 3**). These proteins are essential for multiple physiological processes in plants, as their reduction limits the synthesis of essential proteins, thereby affecting photosynthesis, hormonal signaling, and oxidative damage repair. Structural and enzymatic proteins may also be impacted, affecting the growth rate of leaves, stems, and roots. Furthermore, the production of key components for photosystems and the electron transport chain may be disrupted. Finally, a decrease in the synthesis of protective and repair proteins makes plants more susceptible to stress.

**Table 3:** List of genes belonging to ribosomal proteins showing the protein name, GO number, and Fold-change.

Genes	Name protein	GO number	Fold-change
VitviT2T_024596	30S ribosomal protein 3, chloroplastic	ENOG5037VMA	-3,393389501
VitviT2T_008642	30S ribosomal protein S1	ENOG5037R7F	-2,105180735
VitviT2T_021073	30S ribosomal protein S1, chloroplastic	ENOG5037QBM	-2,154929133
VitviT2T_023929	30S ribosomal protein S20, chloroplastic	ENOG5037TQK	-2,295679459
VitviT2T_029516	30S ribosomal protein S9, chloroplastic	ENOG5037K1X	-2,791590649
VitviT2T_029719	40S ribosomal protein S23	ENOG5037SQP	2,02644679
VitviT2T_030312	50S ribosomal protein L12, chloroplastic	ENOG5037U56	-2,201922199
VitviT2T_018264	50S ribosomal protein L13, chloroplastic	ENOG5037QQI	-2,099333721
VitviT2T_017552	50S ribosomal protein L21, chloroplastic	ENOG5037U92	-2,009040705
VitviT2T_006164	50S ribosomal protein L27, chloroplastic	ENOG5037IPF	-2,451874831
VitviT2T_023808	50S ribosomal protein L35	ENOG5037V64	-2,277315583
VitviT2T_015683	50S ribosomal protein L35	ENOG5037V64	-2,543510053
VitviT2T_009204	Large ribosomal subunit protein bL17c	ENOG5037HJC	-2,181319209
VitviT2T_014565	Large ribosomal subunit protein bL20c	ENOG5037ZW0	-2,820677406
VitviT2T_013423	Large ribosomal subunit protein bL28c	ENOG5037VFT	-2,907666835
VitviT2T_017483	Large ribosomal subunit protein bL9c	ENOG5037IT5	-2,487487726
VitviT2T_023302	Large ribosomal subunit protein uL15/eL18 domain-containing protein	ENOG5037P2T	-2,150988538
VitviT2T_011261	Large ribosomal subunit protein uL15/eL18 domain-containing protein	ENOG5037TS4	-3,997014902
VitviT2T_000282	Large ribosomal subunit protein uL29c	ENOG5037UFQ	-2,003220779
VitviT2T_017909	Large ribosomal subunit protein uL5c	ENOG5037HPD	-2,225018095
VitviT2T_028465	Large ribosomal subunit protein uL6 alpha-beta domain-containing protein	ENOG5037NEG	-2,393716036
VitviT2T_029364	Small ribosomal subunit protein bS6c	ENOG5037TV5	-2,358369369
VitviT2T_007580	Small ribosomal subunit protein uS17c	ENOG5037UH4	-2,350610343
VitviT2T_020527	Small ribosomal subunit protein uS19c	ENOG5037WJH	-3,416313143
VitviT2T_007966	Small ribosomal subunit protein uS7cz/uS7cy	ENOG5037JZ5	-2,82476673

#### 3.3.2 Effect of LEE on *Vitis vinífera* plants

**Figure 4:**
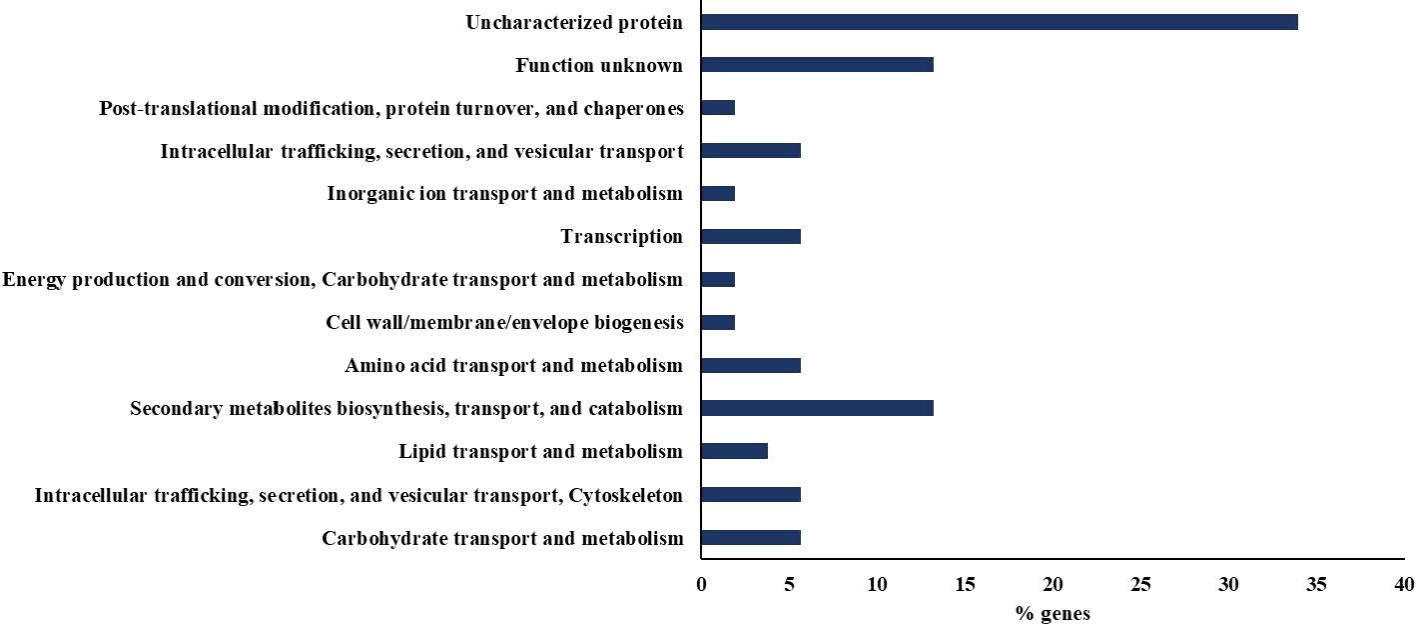
Distribution of the percentage of differentially expressed genes after exposure to LEE classified into functional categories assigned by COG.

The application of the hydrolysate from red wine lees caused 64 genes to be differentially expressed, of which 41 were repressed and 23 were overexpressed compared to the control plants. The statistical analysis supporting these results is provided in the Supplementary Material (**Supplementary Figures S6**-**S10**). As observed in **Figure 4**, around 34% of the genes were annotated as Uncharacterized protein and 13% as Function unknown. The most representative category by the number of genes was Secondary metabolites biosynthesis, transport, and catabolism with 13%, being overexpressed certain proteins important for plant metabolism, for example, the Fe2OG dioxygenase domain-containing proteins (VitviT2T_027113 and VitviT2T_027883), which are involved in the biosynthesis of flavonoids, and the Phenylalanine ammonia-lyase (VitviT2T_007942), Key enzyme in the phenylpropanoid pathway. These results suggest that the overexpression of these genes induced by the biostimulant may improve the plant’s ability to defend itself, adapt, and produce valuable compounds, although it could also imply a metabolic cost that might affect growth, for example.

It’s worth noting that the application of the biostimulant has led to the overexpression of the glutathione transferase (VitviT2T_005150), A key enzyme in the metabolism of *Vitis vinifera*, as it plays a crucial role in detoxification functions, maintaining redox balance, and responding to abiotic and biotic stress, by catalyzing the conjugation of glutathione with a wide variety of toxic compounds, facilitating their neutralization and elimination (Nianiou-Obeidat et al., 2017).

#### 3.3.3 Effect of LEE on plants stressed with 300 ppb of ozone

Next, the effect of the biostimulant on plants stressed by 300 ppb of ozone was studied. For this purpose, the 4,092 differentially expressed genes produced in the presence of 300 ppb of ozone were compared with the 240 differentially expressed genes in ozone-affected plants to which the biostimulant was applied. The statistical analyses associated with these comparisons are provided in the Supplementary Material (**Supplementary Figures S11**-**S15**).

**Figure 5:**
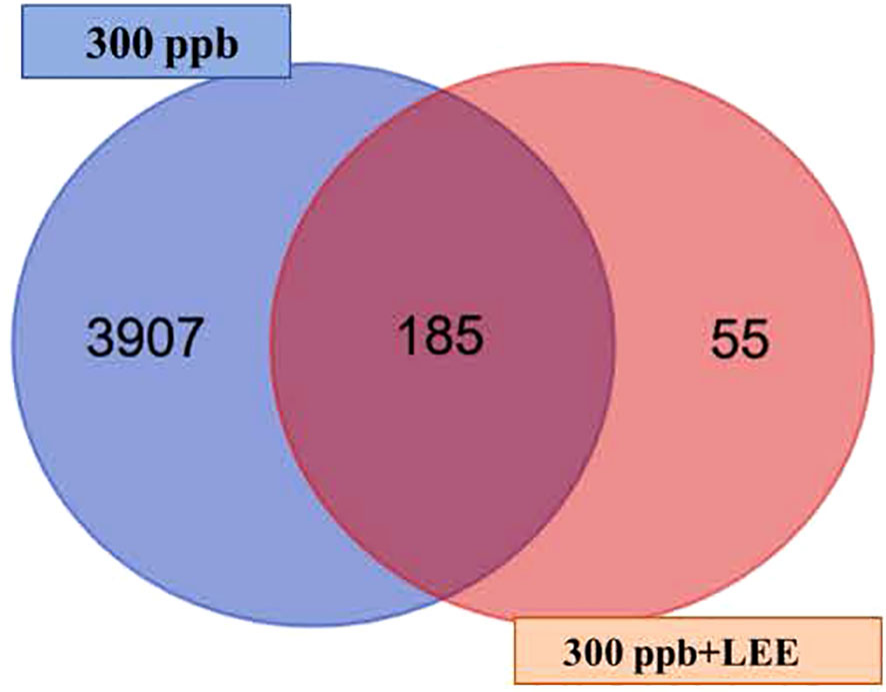
Venn diagram showing the differentially expressed genes in plants exposed to 300 ppb of ozone (purple) and the differentially expressed genes in plants exposed to ozone and LEE (orange).

As observed in the Venn diagram (**Figure 5**), the control plants (300 ppb) and the plants treated with ozone plus LEE shared 185 common genes and 55 genes that are only expressed in the presence of LEE.

A more detailed study of these 185 genes has shown that, in the presence of ozone, a set of proteins related to the plant’s protection against ozone-induced stress were overexpressed. When the biostimulant was applied, the FoldChange of these genes changed drastically, being less overexpressed (an example is chlorophyll a-b binding proteins) or even leading to gene repression (stilbene synthases) (**Table 4**).

**Table 4:** Set of differentially expressed genes in both ozone-treated plants (FoldChange 300 ppb) and ozone plus LEE-treated plants (FoldChange 300 ppb + LEE).

Function	ID	FoldChange 300 ppb	FoldChange 300 ppb + LEE	Name protein
Energy production and conversion	VitviT2T_014996	30,1632005	7,133692838	Chlorophyll a-b binding protein, chloroplastic
	VitviT2T_016247	101,9236827	3,142235122	Chlorophyll a-b binding protein, chloroplastic
	VitviT2T_007236	10,24617636	-45,07296606	Glycosyltransferase
	VitviT2T_001779	3,792418645	-4,261955483	Glycosyltransferase
Post-translational modification, protein turnover, and chaperones	VitviT2T_012202	1734,27786	-4,450647785	glutathione S-transferase L3
	VitviT2T_008238	17,51724572	-9,936281563	glutathione transferase
	VitviT2T_012355	16,86216849	-5,365817131	glutathione transferase
	VitviT2T_030419	20,18812354	-5,56253065	glutathione transferase
Coenzyme transport and metabolism	VitviT2T_025030	61,19453714	-29,07807299	Stilbene synthase 3
	VitviT2T_025014	140,4405506	-12,13113083	Stilbene synthase 5
	VitviT2T_025019	2,63003141	-22,87618799	trihydroxystilbene synthase
	VitviT2T_025038	55,6265669	-14,28655465	trihydroxystilbene synthase
	VitviT2T_025033	126,0270103	-14,05213102	trihydroxystilbene synthase
Transcription	VitviT2T_006696	-2,411114	-33,32433559	ethylene-response factor C3
	VitviT2T_025589	32,22842737	-6,427397331	ethylene-responsive transcription factor ERF113
	VitviT2T_015089	18,04645259	-6,517067766	transcription factor bHLH162
	VitviT2T_029639	286,3906312	-5,649754386	Transcription factor MYB102
	VitviT2T_004493	13,59187959	-3,383466533	transcription factor MYB123
	VitviT2T_025872	21,86392555	-5,663569672	transcription factor MYB62
	VitviT2T_004497	5,420384393	-20,63127176	Transcription factor WER
	VitviT2T_009629	3,1177692	-2,781755389	WRKY transcription factor
Carbohydrate transport and metabolism	VitviT2T_029896	42,35488504	-17,49049236	glucan endo-1,3-beta-D-glucosidase
	VitviT2T_008906	47,30300039	-3,81969279	glucan endo-1,3-beta-glucosidase-like
Amino acid transport and metabolism	VitviT2T_026076	46,2481922	-3,189784151	protein NRT1/PTR FAMILY 2.11
	VitviT2T_000875	6,031526889	-2,834455899	Protein NRT1/PTR family 4.6
	VitviT2T_023196	2,908580453	-6,53022495	Protein NRT1/PTR family 7.3

The table shows the function, gene identity (ID), FoldChanges, and protein names.

A set of proteins stood out as being overexpressed when LEE was present compared to the control, where they were repressed (VitviT2T_024155, VitviT2T_018696, VitviT2T_013309). These genes are probable leucine-rich repeat receptor-like protein kinase At1g35710, which play fundamental roles in signal perception and transduction.

It is noteworthy that, of the 55 differentially expressed genes in plants treated with LEE, 5 were found to be repressed while the rest were overexpressed. It was observed that the signal transduction mechanisms in these plants are overstimulated, as several LRR receptor-like serine/threonine-protein kinases were found to be overexpressed. These kinases are essential for regulating growth, adapting to the environment, and defending these plants. Therefore, this could have potential applications in improving grapevine crops to make them more resistant and productive.

## 4 Discussion

The grapevine plays a fundamental role in the cultural, economic, and ecological aspects of the Mediterranean environment (Moura et al., 2023). Globally, this crop holds great economic importance (Blanco-Ward et al., 2021), and wine production is a key activity that is an integral part of the cultural identity of many countries (Ascenso et al., 2021).

The ability of tropospheric ozone to damage vegetation is widely studied, manifesting in reduced photosynthesis, accelerated cellular aging, increased susceptibility to diseases, decreased growth, and reduced reproductive capacity in plants. In the specific case of *Vitis vinifera*, it has been observed that it exhibits intermediate sensitivity to ozone (Ascenso et al., 2021).

The present study demonstrates the protective effect of a biostimulant (LEE) derived from red wine lees against ozone-induced damage in grapevine plants. The analysis of LEE composition reveals that it is a potential source of active molecules such as proteins and phenols (**Table 1**). The use of proteases enables the release of peptides from proteins, converting them into their active form (Justus et al., 2019). The resulting protein hydrolysates exhibit various biological functions, with antioxidant activity being one of the first recognized (Zhu et al., 2022).

Due to these properties, are considered an innovative alternative for stimulating plant growth. Their foliar application can help plants cope with abiotic stress by enhancing their antioxidant capacity (Sitohy et al., 2020). LEE also provides essential amino acids (threonine, valine, isoleucine, and leucine) that plants cannot synthesize and must obtain from the biostimulant (Sun et al., 2024).

The high content of phenolic compounds in the biostimulant further highlights its potential as an effective tool for enhancing plant health, as these compounds play a key role in plant development and defense mechanisms. Their multifaceted action significantly contributes to plant resilience and adaptation to adverse environmental conditions. Moreover, phenolic compounds are involved in the regulation of various signaling pathways associated with stress responses, underscoring their essential function in coordinating plant defense systems (Saini et al., 2024). Additionally, they have other applications in bioremediation and as antioxidants in food additives (Sharma et al., 2019).

The ozone treatment significantly affects the physiological state of the plants, causing a decrease in photosynthetic parameters, including A_N_, ETR, PhiPII, and delayed fluorescence. The biostimulant treatment completely reversed these effects (**Figures 1A-C**; **Figures 2A-B**). Therefore, we propose that LEE may exert a biostimulant effect on grapevine plants by improving their overall physiological state.

Ozone-induced damage in living organisms is primarily due to its high oxidizing power. This damage mainly results from the generation of reactive oxygen species (ROS), either through the spontaneous decomposition of O_3_ upon entering the apoplastic space or through direct interactions with various cellular components. These processes trigger oxidative damage to biomolecules, potentially compromising crucial cellular functions (Agathokleous et al., 2015).

Accordingly, the protective effect of LEE in plants exposed to ozone can be largely attributed to the antioxidant capacity of the biostimulant, as demonstrated by the DPPH and ABTS values (**Table 1**), a particularly relevant aspect to highlight. Another notable characteristic of this product is its osmoprotective ability, which is due to the high amount of the amino acid proline present in the hydrolysate (**Table 2**). According to Raza et al. (2023), plants accumulate this amino acid in response to various abiotic stresses. In addition to helping maintain osmotic balance, proline preserves cell turgor and indirectly modulates the metabolism of reactive oxygen species (ROS).

Currently, the advancement of omics sciences, driven by the development of genome sequencing technologies and the reduction in their costs, has transformed the understanding and identification of metabolic pathways in plants (Owen et al., 2017). To evaluate the effect of ozone and the biostimulant on plants, a transcriptomic analysis was performed using the RNA-Seq technique, allowing us to analyze global gene expression changes with high precision. The RNA-Seq analysis reveals significant changes in gene expression in response to ozone and the biostimulant treatment.

Compared to control plants, those exposed to ozone exhibit inhibited 50S and 30S ribosomal proteins present in chloroplasts and mitochondria (**Table 3**). These findings suggest a substantial impairment in the synthesis of proteins essential for key cellular processes such as photosynthesis, respiration, and antioxidant defense, ultimately compromising plant cell health and viability. Consistently, previous studies conducted on Arabidopsis thaliana exposed to elevated ozone concentrations (≥300ppb) reported a significant downregulation of genes encoding chloroplast ribosomal subunits (e.g., tAPX and Cu/Zn SOD, both associated with antioxidant pathways) and mitochondrial components (e.g., Mn SOD), thereby corroborating our results (Li et al., 2006).

Genes related to oxidative stress are differentially expressed, including transcription factors associated with phytohormones, highlighting, for example the ethylene-responsive transcription factors. Ethylene production, which can occur in high amounts following ozone exposure, may lead to cell death; however, ethylene at low concentrations can induce defense genes in *Arabidopsis* (Tamaoki et al., 2003). Proteins containing the BHLH (Basic Helix-Loop-Helix) domain were also found, as well as MYB, WRKY, and NAC transcription factors. Additionally, there was an overexpression of proteins that function as defense and adaptation mechanisms to minimize damage to proteins and other cellular components (Asthir, 2015; Foyer and Noctor, 2005). These include post-translational modifications, protein turnover, and chaperones, as well as the overexpression of glutathione S-transferases, oxidoreductases. These results align with findings in tomato, wheat, and barley plants (Gallé et al., 2009; Rezaei et al., 2013), where high expression of these enzymes correlated with greater tolerance to abiotic stress. This acclimation could also be due to redox regulations exerted by thioredoxins and glutaredoxins, which were differentially expressed upon ozone application. These two oxidoreductases regulate signal transduction pathways associated with plant growth, defense, and productivity (Jiménez et al., 2024).

The overexpression of pathways involved in the synthesis of biotin and phenolic compounds observed when plants are exposed to ozone provides valuable insights. On one hand, the activation of phenolic pathways (such as phenylpropanoids, stilbenes, and flavonoids) leads to the production of secondary metabolites associated with antioxidant activity, pathogen defense, and environmental stress resistance, as well as the strengthening of the cell wall. On the other hand, biotin is essential for fatty acid metabolism, gluconeogenesis, and abiotic stress responses, acting as a protective molecule (Cataldo et al., 2022; Noronha et al., 2023). These effects have been observed with the use of *Ascophyllum nodosum* (a brown seaweed) extracts, which stimulate antioxidant enzymes and phenylpropanoid pathways (including resveratrol) in tomato, enhancing defense against pathogens such as *Botrytis* and *Plasmopara* (Kanojia et al., 2024). Additionally, in *Vitis vinifera*, biostimulants have been shown to increase the accumulation of phenolics, biotin, and defense-related enzymes, thereby improving, for instance, drought tolerance (Irani et al., 2021). These pathways are crucial for wine quality and human health (Reinisalo et al., 2015).

The application of the biostimulant in ozone-stressed plants demonstrates that this product could mitigate stress by reducing oxidative damage in these plants while also enhancing resilience, as the plant may be better prepared to defend itself. This is evidenced by the overexpression of flavonoid and phenylpropanoid biosynthesis pathways in the presence of LEE (Bulgakov et al., 2024; Dao et al., 2025).

Another important aspect is resource conservation for the plant, as there is lower energy expenditure on defense due to the reduced activation of emergency stress responses. As shown in **Table 4**, the FoldChange of stress-related genes is less overexpressed or even repressed. Additionally, differentially expressed genes were found which play fundamental roles in signal perception and transduction. Studies have shown that the overexpression of these genes in *Vitis vinífera* model plants improves drought tolerance, indicating their potential for genetic improvement of crops under adverse conditions (Liu et al., 2024).

Therefore, the biostimulant alleviates the impact of ozone, optimizing metabolic efficiency and plant health.

## 5 Conclusion

In this study, we have analyzed the protective effect of a biostimulant obtained from red wine lees against abiotic stress induced by ozone in *Vitis vinifera* plants. Ozone has negative effects on both the growth and overall health of grapevines, which can lead to a decrease in productivity and wine quality. This is particularly concerning in areas where ozone concentrations are high due to atmospheric pollution.

The application of LEE effectively reversed the negative effects of ozone on plant physiology, improving photosynthetic activity and overall plant health. The antioxidant and osmoprotective properties of the biostimulant, along with its ability to influence key metabolic pathways, provide significant resilience against oxidative stress. In addition, the biostimulant optimizes resource use in plants by reducing the activation of emergency stress responses, thus supporting their growth and productivity. These results indicate that LEE may represent a valuable strategy to obtain a functional biostimulant from viticulture by-products, contributing to the circular use of grape-derived residues.

A limitation of this study is that all experiments were conducted under controlled greenhouse conditions on potted *Vitis vinifera* cv. Syrah plants, rather than in commercial vineyards. Consequently, the responses observed here may not fully represent the performance of grapevines grown under open-field conditions, and further trials in commercial vineyards will be necessary to validate the effectiveness and agronomic relevance of this biostimulant.

## Data Availability

The original contributions presented in the study are publicly available. This data can be found here: https://www.ncbi.nlm.nih.gov/, accession number PRJNA1377885.
