# Supplementary material for: A novel biostimulant obtained from red wine lees enhances tolerance to ozone-induced abiotic stress in grapevine plants
Source: Front Plant Sci. 2026 Feb 3;17:1654494. doi: 10.3389/fpls.2026.1654494 (PMC12910366; doi:10.3389/fpls.2026.1654494)
Supplement: Supplementary file 1 [file DataSheet1.docx]

***Supplementary Material***

**A novel biostimulant obtained from red wine lees enhances tolerance to ozone-induced abiotic stress in grapevine plants**

E. Naranjo^*^, A. Orts, J.M. Orts, L. Martin-Presas, A. Castaño, J. Parrado

Departament of Biochemistry and Molecular Biology, Facultad de Farmacia, Universidad de Sevilla, C/Prof. García González 2, 41012 Sevilla, Spain

**Corresponding*: E. Naranjo: [eminaranjo@us.es](mailto:eminaranjo@us.es)

**1 Supplementary Figures**


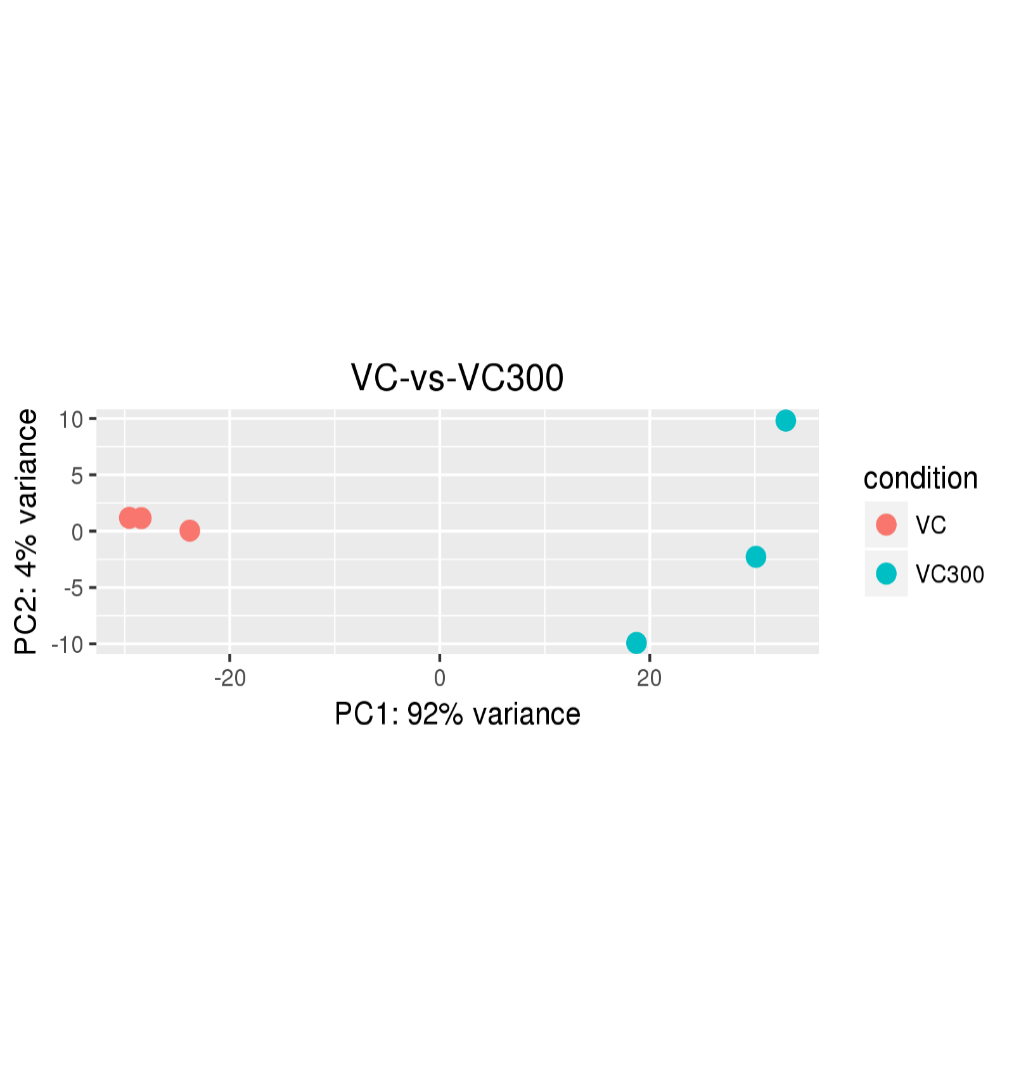


**Supplementary Figure S1.** Principal Component Analysis (PCA) showing the separation between control plants (VC) and plants exposed to 300 ppb ozone (VC300). PC1 explains 92% of the total variance and clearly discriminates between the two groups, while PC2 accounts for the remaining 4%. Each point represents an individual sample according to its experimental condition.


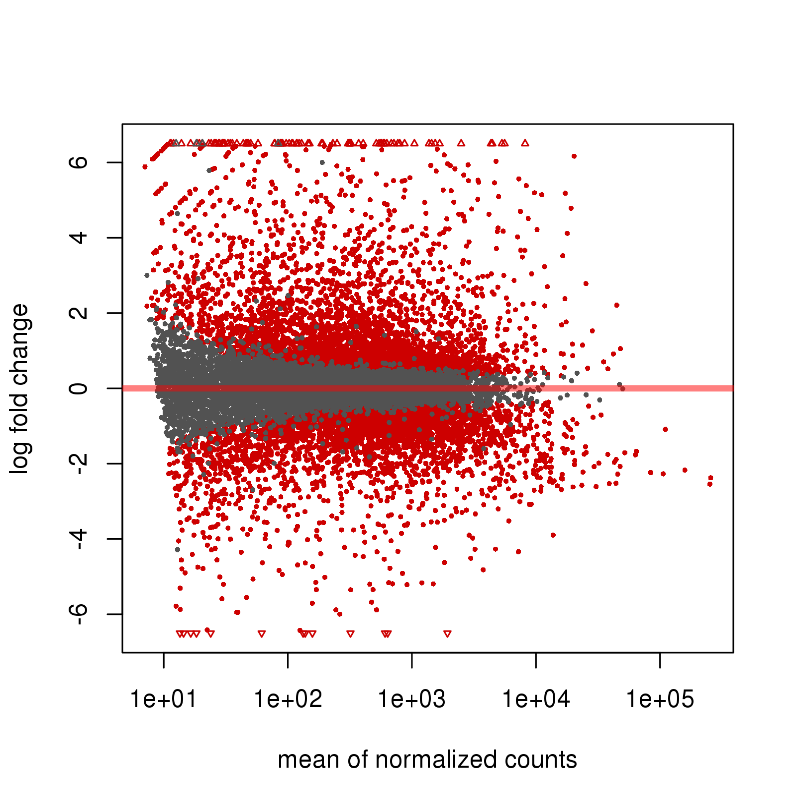


**Supplementary Figure S2.** MA-plot depicting the relationship between the mean of normalized counts and the log fold change for all analyzed genes, comparing control plants with plants exposed to 300 ppb ozone. Red points indicate genes with statistically significant differential expression, whereas gray points represent genes without statistically significant changes.

**
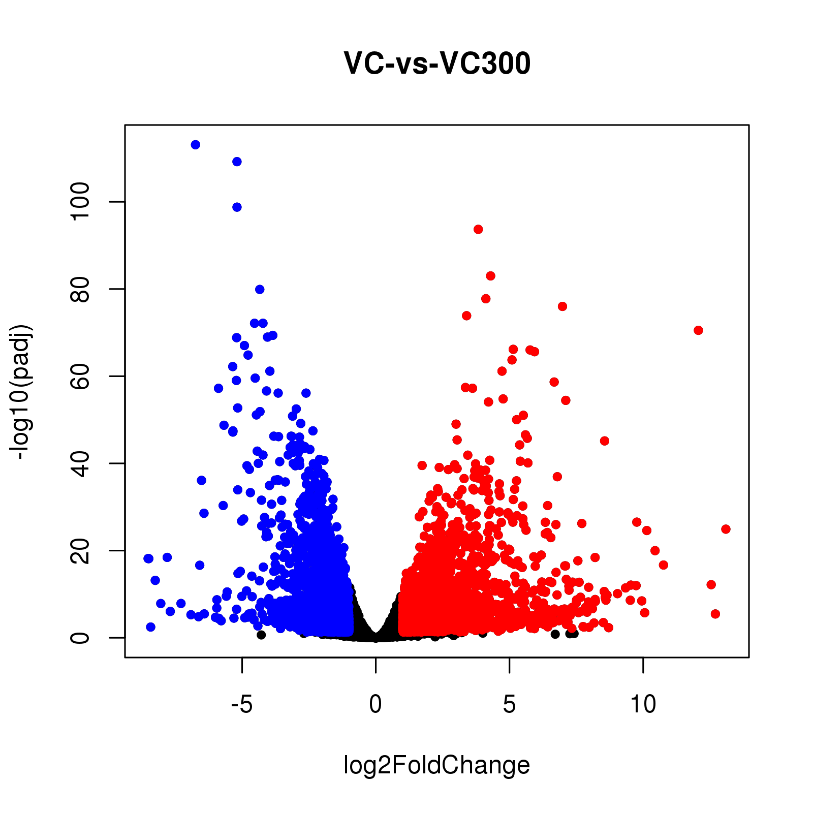
**

**Supplementary Figure S3.** Volcano plot illustrates the distribution of differentially expressed genes when comparing control plants (VC) to plants exposed to 300 ppb ozone (VC300). The x-axis represents the log₂ fold change, while the y-axis displays the –log₁₀(adjusted p-value). Red points correspond to significantly upregulated genes, blue points indicate significantly downregulated genes, and black points represent genes without statistically significant changes.

**
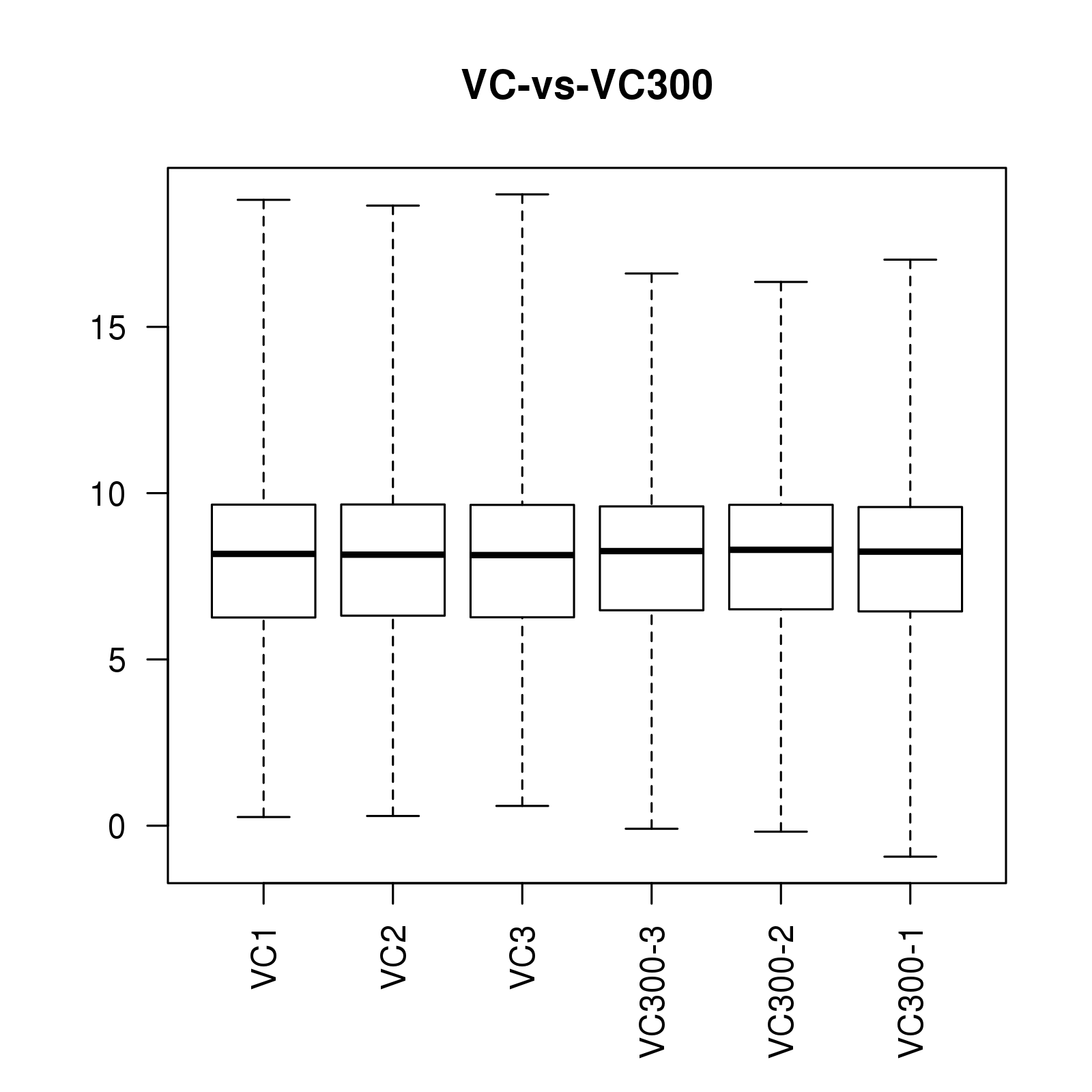
**

**Supplementary Figure S4.** Distribution of the response variable in control plants (VC1–VC3) and plants exposed to ozone at 300 ppb (VC300-3, VC300-2, and VC300-1), shown as boxplots indicating the median, interquartile range, and extreme values for each experimental group under acute ozone exposure.


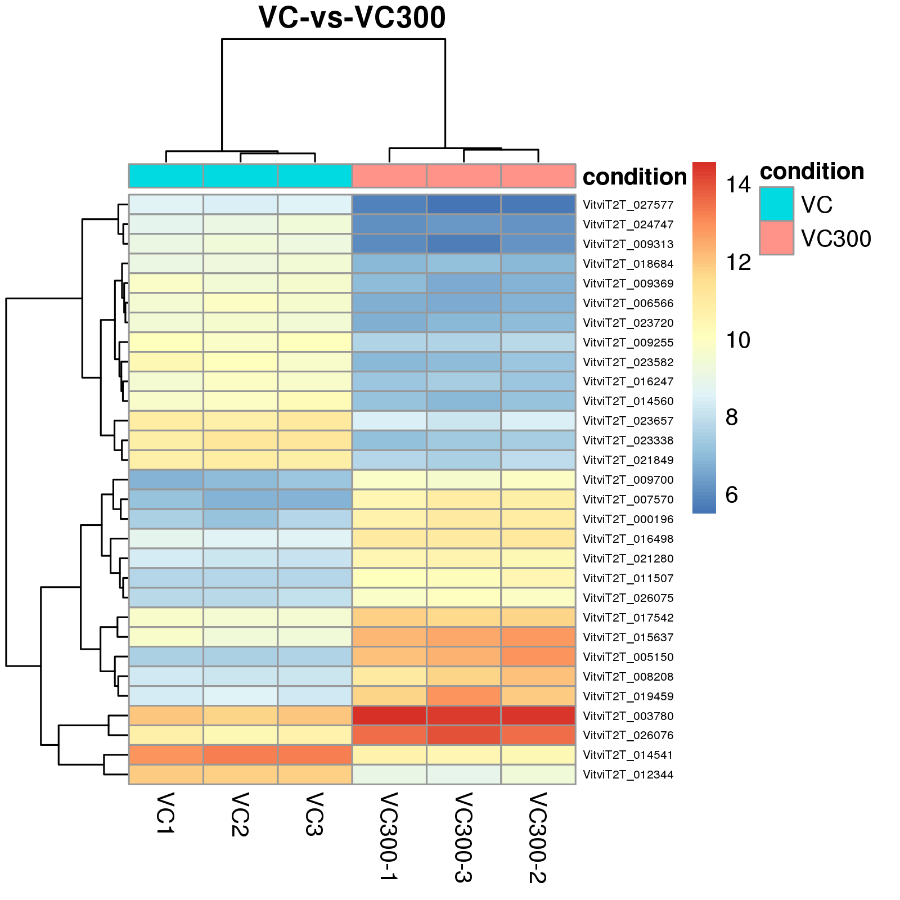


**Supplementary Figure S5.** Biclustering analysis of the top differentially expressed genes (selected by adjusted p-value) comparing control plants (VC) and plants exposed to 300 ppb ozone (VC300).


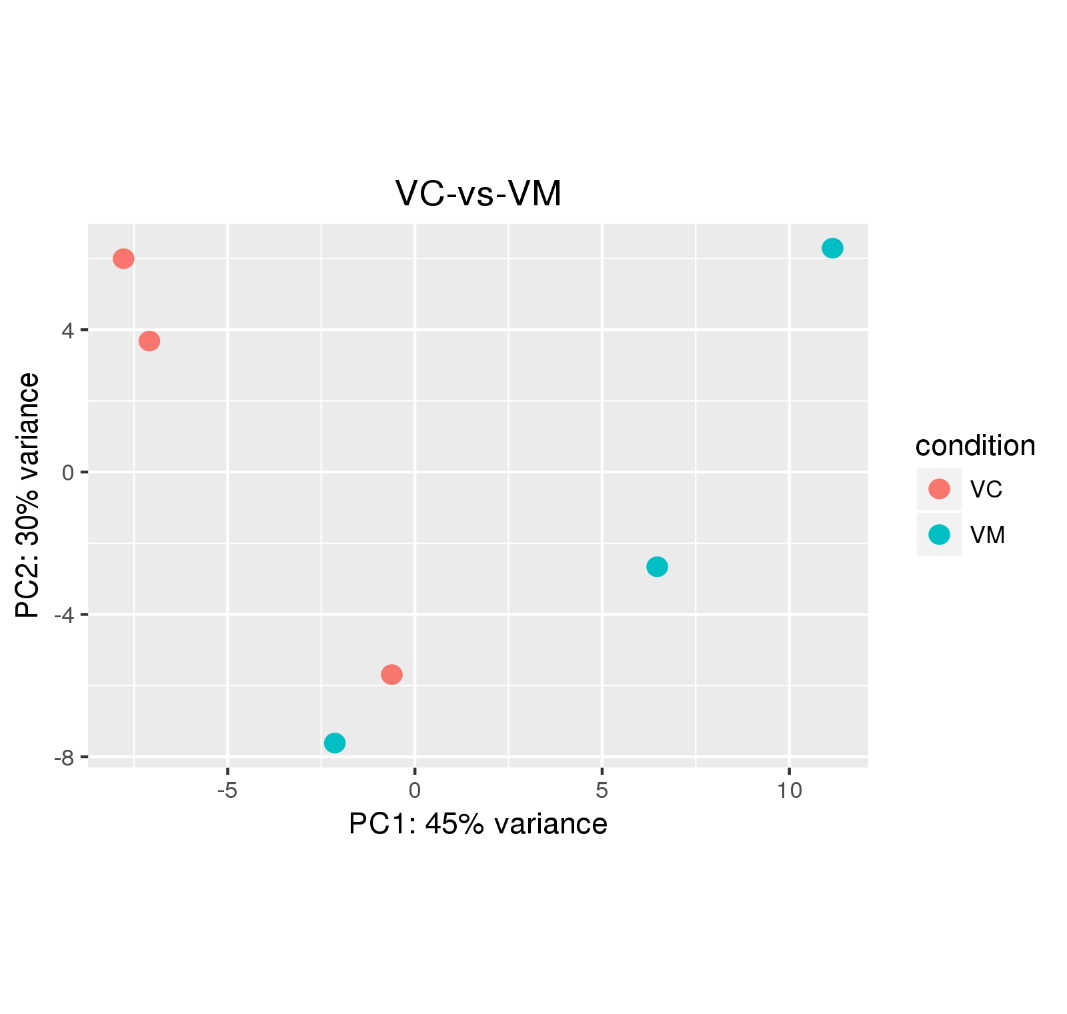


**Supplementary Figure S6.** Principal component analysis (PCA) depicting the distribution of samples from control plants (VC, red circles) and LEE-treated plants (VM, blue circles) along the first principal component (PC1, 45% of the explained variance) and the second principal component (PC2, 30% of the explained variance).


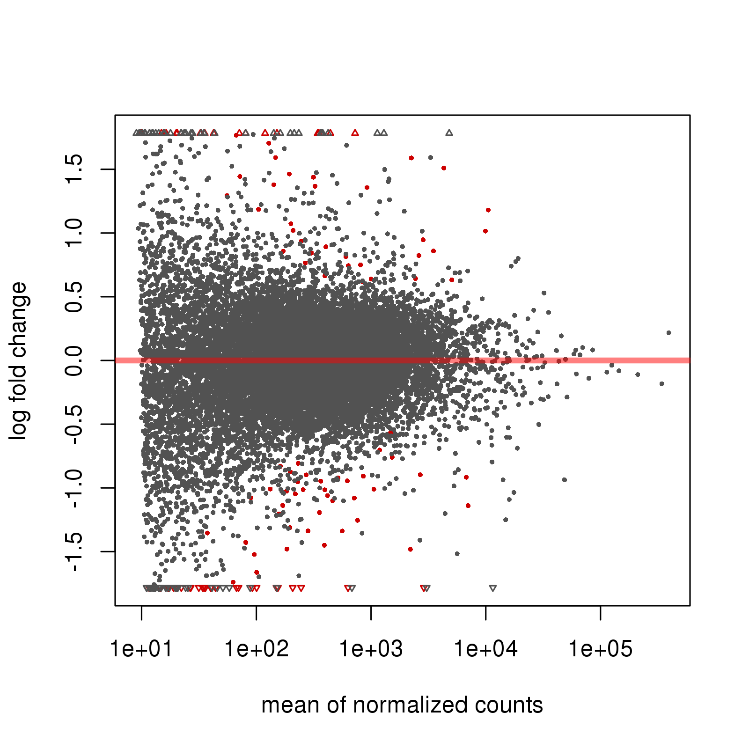


**Supplementary Figure S7.** MA-plot depicting the relationship between the mean of normalized counts and the log fold change for all analyzed genes, comparing control plants with plants exposed to LEE. Red points indicate genes with statistically significant differential expression, whereas gray points represent genes without statistically significant changes.


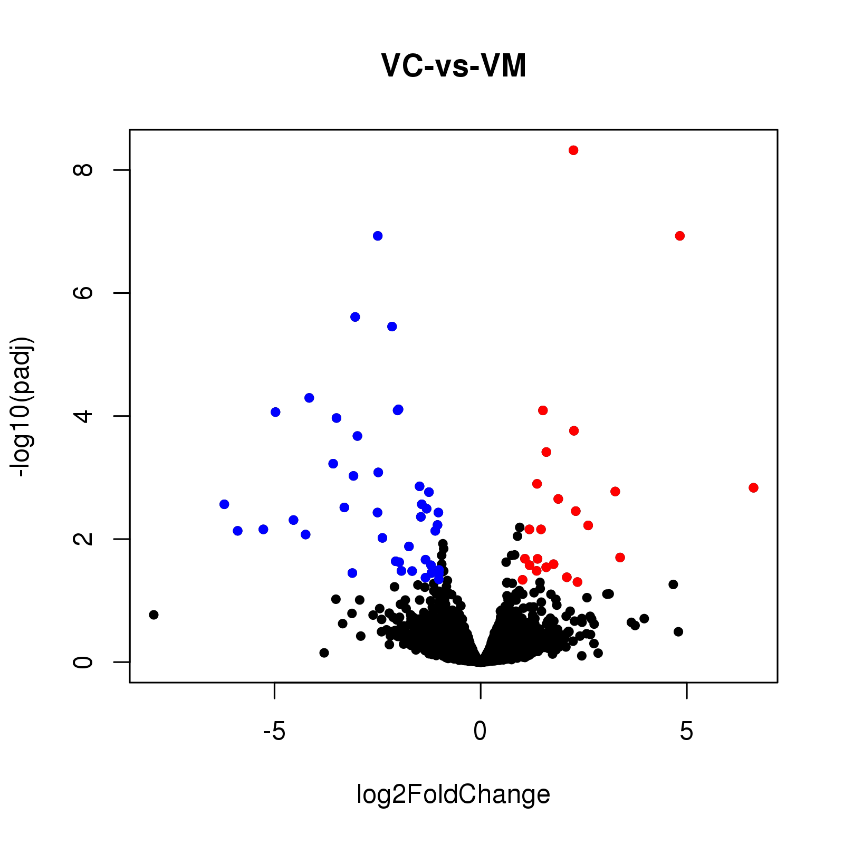


**Supplementary Figure S8.** Volcano plot illustrating the distribution of differentially expressed genes when comparing control plants (VC) to plants exposed to LEE (VM). The x-axis represents the log₂ fold change, while the y-axis displays the –log₁₀(adjusted p-value). Red points represent significantly upregulated genes, blue points indicate significantly downregulated genes, and black points represent genes without statistically significant changes..


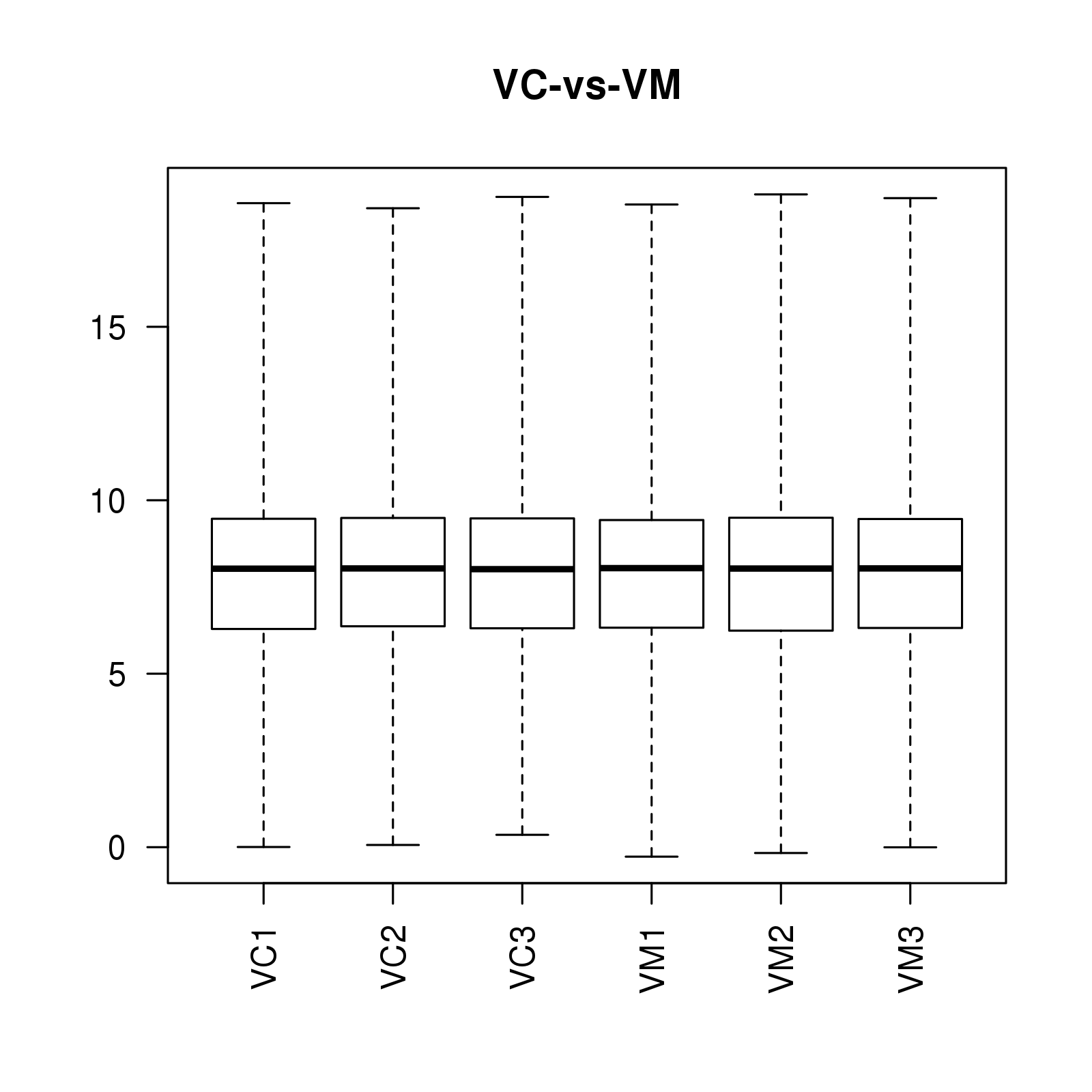


**Supplementary Figure S9.** Distribution of the response variable in control plants (VC1–VC3) and plants exposed to LEE (VM1, VM2, and VM3), shown as boxplots indicating the median, interquartile range, and extreme values for each experimental group under acute ozone exposure.


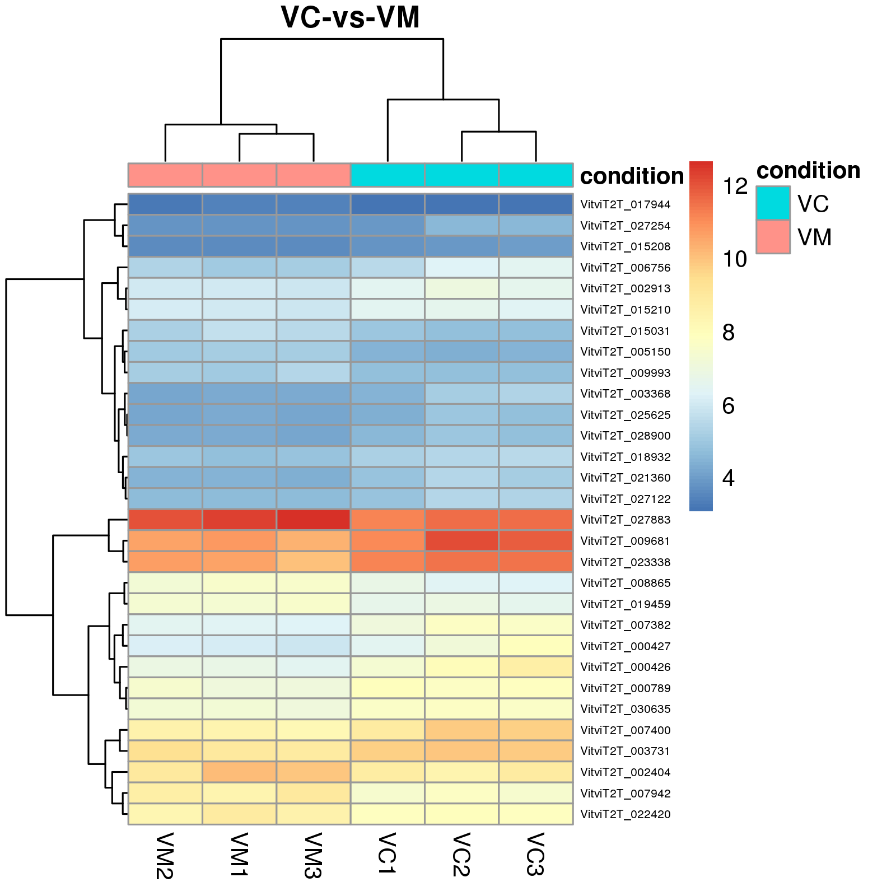


**Supplementary Figure S10.** Biclustering analysis of the top differentially expressed genes (selected by adjusted p-value) comparing control plants (VC) and plants exposed to LEE (VM).


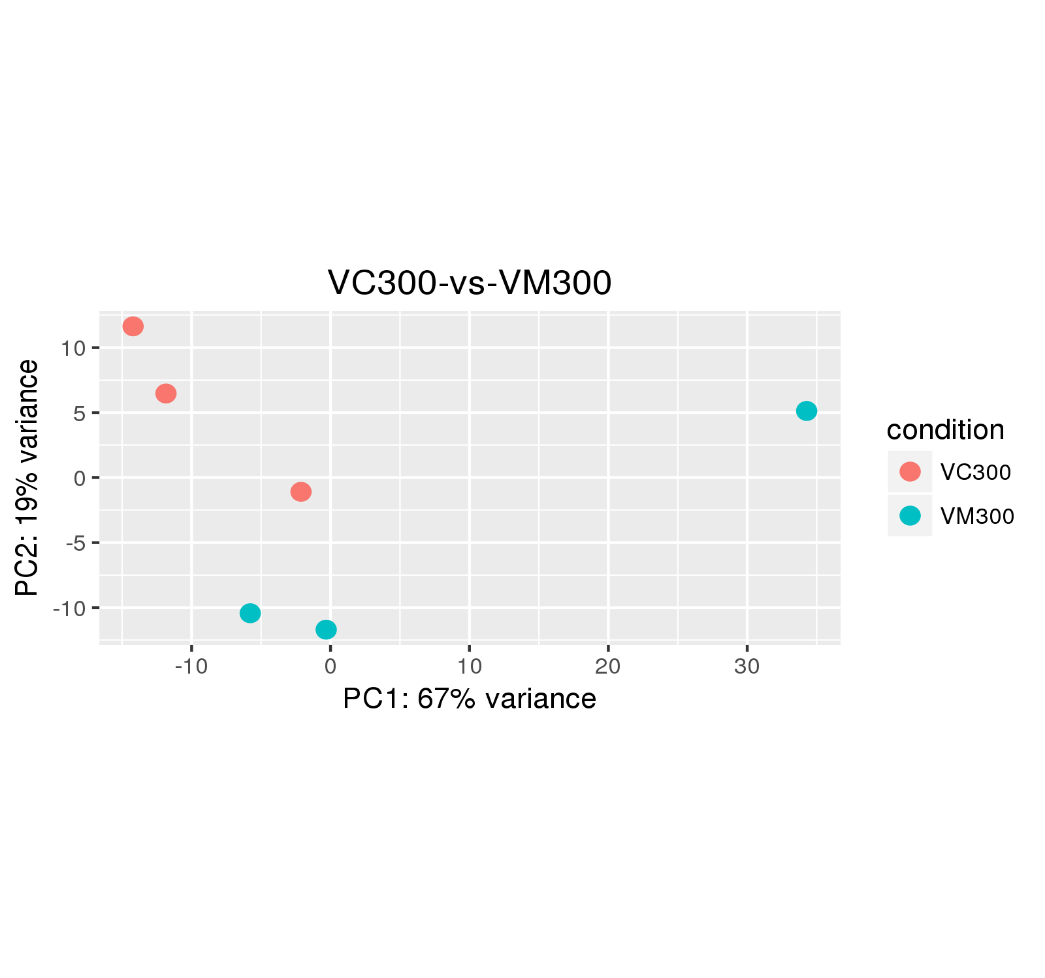


**Supplementary Figure S11.** Principal component analysis (PCA) depicting the distribution of samples from plants exposed to 300 ppb of ozone (VC300, red circles) and LEE+Ozone-treated plants (VM300, blue circles) along the first principal component (PC1, 67% of the explained variance) and the second principal component (PC2, 19% of the explained variance).


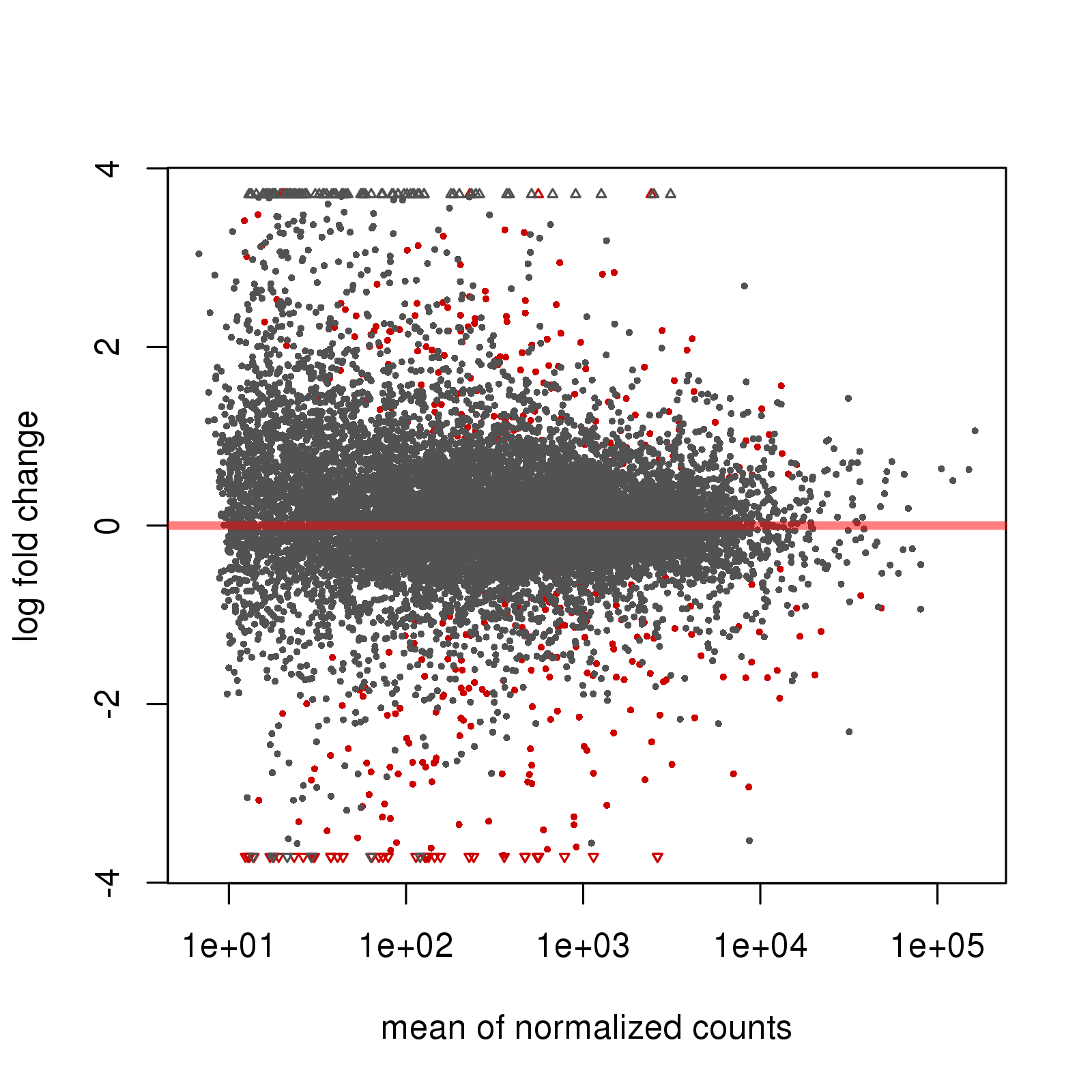

**Supplementary Figure S12.** MA-plot depicting the relationship between the mean of normalized counts and the log fold change for all analyzed genes, comparing plants exposed to ozone (300ppb) with plants exposed to LEE + 300 ppb ozone. Red points indicate genes with statistically significant differential expression, whereas gray points represent genes without statistically significant changes.


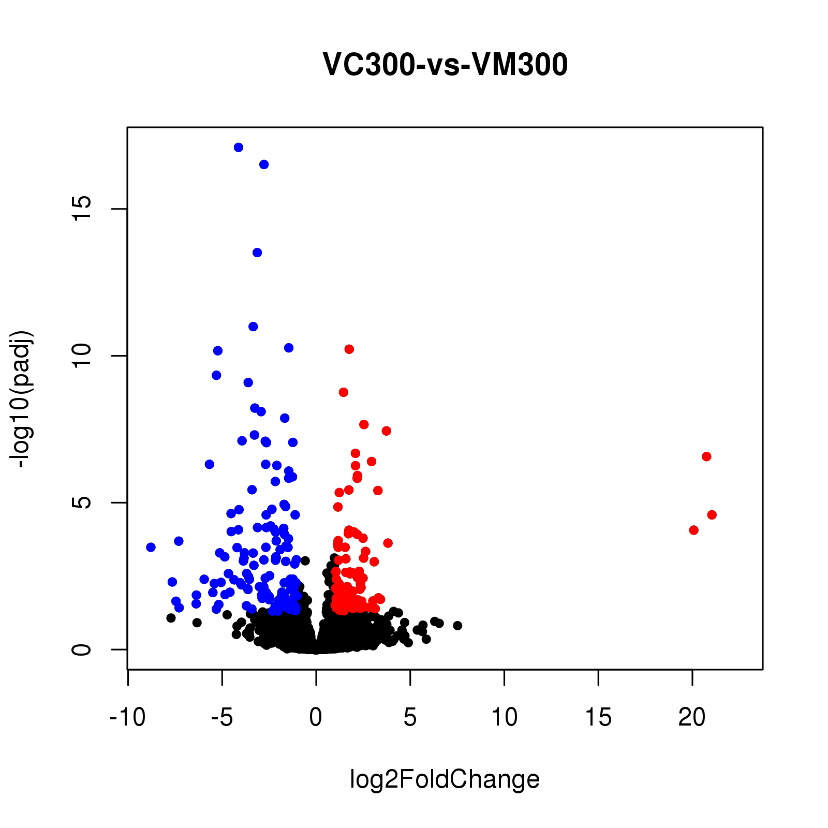


**Supplementary Figure S13.** Volcano plot illustrating the distribution of differentially expressed genes when comparing plants

(VC300) to plants exposed to LEE +Ozone (VM300). The x-axis represents the log₂ fold change, while the y-axis displays the –log₁₀(adjusted p-value). Red points represent significantly upregulated genes, blue points indicate significantly downregulated genes, and black points represent genes without statistically significant changes.


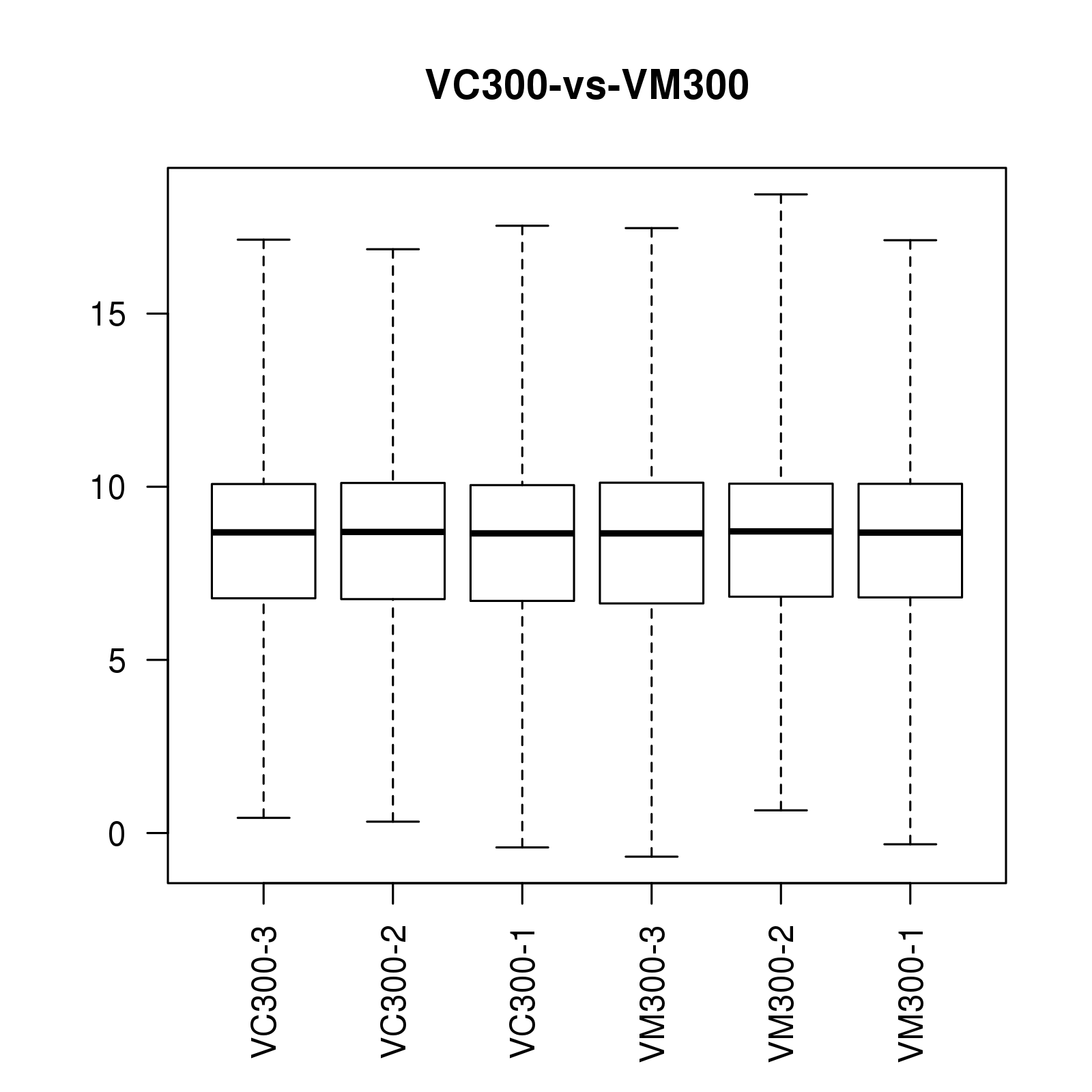


**Supplementary Figure S14.** Distribution of the response variable in plants exposed to ozono (VC300-1–VC300-3) and plants exposed to LEE+ ozone (VM300-1, VM300-2, and VM300-3), shown as boxplots indicating the median, interquartile range, and extreme values for each experimental group under acute ozone exposure.

**
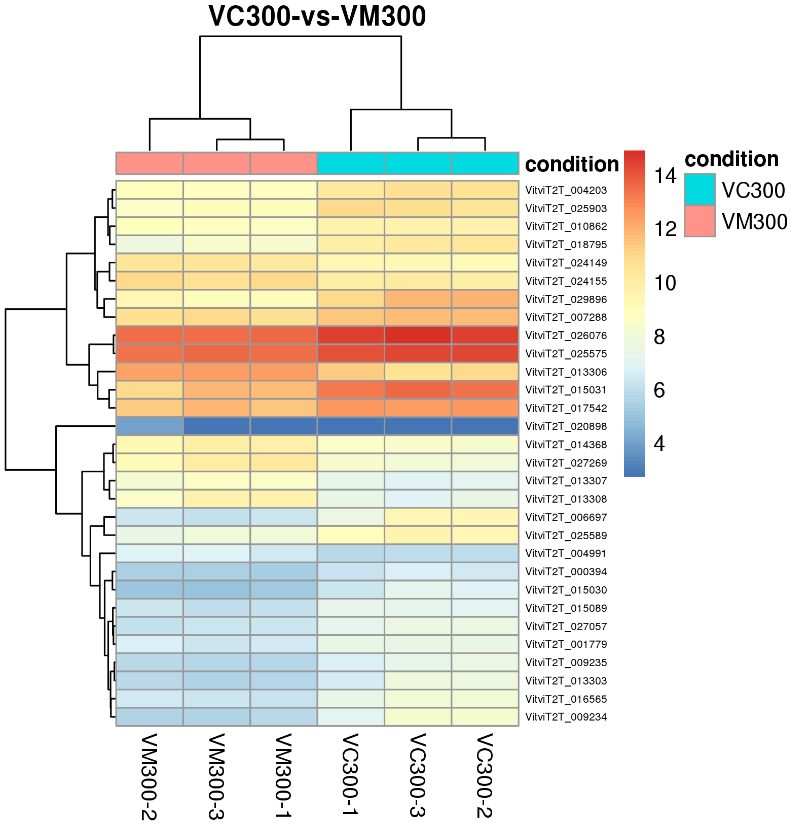
**

**Supplementary Figure S15.** Biclustering analysis of the top differentially expressed genes (selected by adjusted p-value) comparing plants exposed to ozone (VC300) and plants exposed to LEE +Ozone (VM300).
